# Exopolysaccharides from Microalgae and Cyanobacteria: Diversity of Strains, Production Strategies, and Applications

**DOI:** 10.3390/md20050336

**Published:** 2022-05-21

**Authors:** Céline Laroche

**Affiliations:** Clermont Auvergne INP, CNRS, Institut Pascal, Université Clermont-Auvergne, F-63000 Clermont-Ferrand, France; celine.laroche@uca.fr; Tel.: +33-473-407-419

**Keywords:** microalgae, exopolysaccharides, composition, biological activities, physico-chemical properties, valorization

## Abstract

Microalgae and cyanobacteria are photosynthetic organisms that can produce/accumulate biomolecules with industrial interest. Among these molecules, EPSs are macromolecular polysaccharidic compounds that present biological activities and physico-chemical properties, allowing to consider their valorization in diverse commercial markets, such as cosmetic, therapeutic, nutraceutic, or hydrocolloids areas. The number of microalgae and cyanobacteria strains described to produce such EPSs has increased in recent years as, among the 256 producing strains gathered in this review, 86 were published in the last 10 years (~33%). Moreover, with the rise of research on microalgae EPSs, a variety of monosaccharides compositions have been discovered, highlighting the versatility of these organisms. If some production strategies can be applied to increase EPS production yields, it appears that case by case studies are needed to promote EPS synthesis by a strain, as many responses exist. This paper proposes an up-to-date state of the art of the diversity of microalgae and cyanobacteria EPS-producing strains, associated to the variability of compositions. The strategies for the production and extraction of the polymers are also discussed. Finally, an overview of the biological activities and physico-chemical properties allow one to consider their use on several commercial markets.

## 1. Introduction

Over recent decades, microalgae and cyanobacteria have gained interest from the scientific community due to their potential use as a bulk material for biofuel production. However, as well as lipids for biofuels, other valuable biomolecules can be extracted from these organisms such as polyunsaturated fatty acids (PUFAs), pigments, proteins, including some enzymes, and polysaccharides. Moreover, these photosynthetic organisms represent a huge taxonomic diversity, allowing the identification of new producing strains and molecules.

Among the high value compounds from microalgae, polysaccharides (PSs) are very promising. PSs are high molecular weight carbohydrates macromolecules present in all living organisms (plant, bacteria, animal, macro-/microalgae), displaying a large diversity of biochemical structures and functions. They can be homopolysaccharides, composed only of a single repeated monosaccharide, or heteropolysaccharides, composed of two or more distinct sugars. Additionally, they can be linear or branched, and can wear different substituents on their backbone, such as methyl or sulfate groups [1,2].

Even though PSs from animals, fungi, and higher plants (including seaweeds) have been described for many years, studies dealing with PSs produced by microalgae and cyanobacteria have only slowly increased since the 1960s, and only a limited number of species have been identified. Despite their industrial interest, they are then still underexploited and limited to high value markets. This could be due to lower production yields, associated to high production costs. However, the development of industrial culture systems, the improvement of downstream processes, and the implementation of biorefineries’ strategies, associated to a better knowledge about factors affecting production, could help in developing the market chain in the near future [3,4]. Based on their localization and function, PSs from photosynthetic microorganisms can be split into three families: (i) structural PSs from cell walls, (ii) storage PSs which are intracellular, and (iii) extracellular PSs. Many microalgae produce and excrete a polysaccharidic gel matrix, often called mucilages, presumably in order to protect them from fluctuations in environmental conditions. This matrix is supposed to provide protection against various biotic and abiotic stresses, such as desiccation, high light fluxes, and so on, to facilitate the fixation of essential elements and micronutrients present in the environment, to contribute to the attachment of the microorganism to a solid substrate, and to also play a role in the formation of colonies for cyanobacteria [1,5,6,7,8,9,10,11].

These EPSs, depending on the species, could be produced along with growth or under specific conditions that could be associated with nutrient depletion, high salinity, high light fluxes, and so on [1,5,12,13,14]. EPSs from microalgae and notably from cyanobacteria, also contribute to the stabilization of soils by the formation of biological soil crusts [15,16,17,18]. Depending on the photosynthetic microorganisms, the polysaccharides can be totally excreted into the surrounding medium or remain more or less tightly bound to the cells. The terminology used for these extracellular PSs is then somehow confusing. Concerning the bound polymers, they are often called capsular polysaccharides (CPSs), bound polysaccharides (BPSs), but also slime or sheath [5,19], whereas the PSs that are released in the medium can be found under the denomination of exopolysaccharides (EPSs), mucilage, or released exopolysaccharides (RPSs). In many cases, microalgae strains produce both RPSs and BPSs, as shown for red microalgae, for which around 50 to 70% of polysaccharides could remain bounded to the cell [20], but this amount could vary depending on the culture conditions. For the specific case of the cyanobacteria, it has been generally admitted that the exocellular PSs are mainly constituted of CPSs, with only a small amount found as RPSs [1,5]. Nevertheless, this aspect is controversial, as for some species, RPS amounts exceeded those of CPSs, and some others did not produce CPS at all [21]. Today, there is still a thin line between these categories and the use of “EPS” is often used to qualify BPSs. Moreover, there is no clear evidence for compositional differences between RPSs and BPSs produced by a strain, suggesting a common origin. The main difference that has been noticed is the molecular weight of the polymers, RPSs having the greater one [22]. It has then been proposed that these PSs are produced following the same metabolic pathway, first as bound polymers, and then progressively released as functions of some operating parameters. This assumption is correlated with the fact that the RPS/BPS ratio increases with the age of the culture.

Some original rheological properties of the microalgal EPSs have been highlighted, allowing them to be considered as new gelling and thickening agents. To date, the market is mainly occupied by PSs from seaweeds and bacteria, but the atypical properties, associated with the decrease of the production costs in the near future, may open them to new applications. Moreover, many studies have been conducted, both in vitro and in vivo, highlighting some biological activities of microalgal EPSs, such as antibacterial, antioxidant, anti-inflammatory, antiparasitic, immunomodulatory, antitumor, or anticoagulant properties. All these interesting features (huge microalgae and cyanobacteria diversity, original structures, physico-chemical properties, and/or biological activities) make these organisms attractive for the valorization of such compounds in various industrial sectors [14,23].

The objective of this chapter is then to provide an up-to-date overview of EPS production by microalgae and cyanobacteria, in terms of the producing strains and composition, on the factors affecting EPS production and composition, on the extraction and purification methods, and finally, on their potent valorization associated to their biological activities or physico-chemical properties.

## 2. Diversity of Producing Strains and Structures

**Figure 1:**
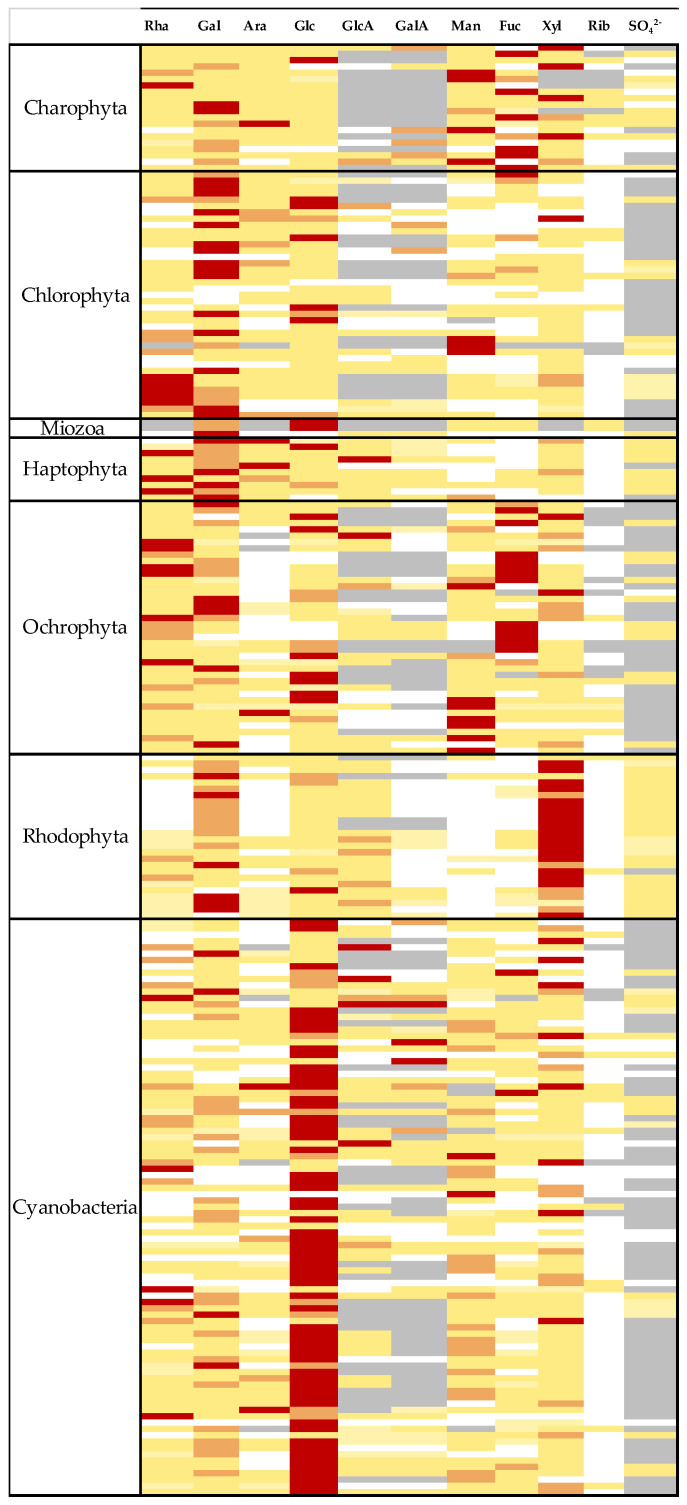
Heat map of EPS compositions. Amounts in each monosaccharide are presented from white (not detected) to red (main monosaccharide). In grey: not evaluated.

The first publication highlighting the production of EPSs by a microalgae was published by Tischer and Moore in 1964 [24], and revealed the monosaccharides composition of the polymer produced from *Palmella mucosa*. Since then, the number of strains identified as producers of EPSs does not cease to increase and can be found in almost all microalgae phyla, such as Charophyta, Chlorophyta, Ochrophyta, miozoa, bacillariophyta, Haptophyta, Rhodophyta [1,25], as well as inside cyanobacteria phylum [26]. However, these described strains are not really correlated with the large taxonomic diversity of microalgae and a huge number of strains have still not been evaluated for EPS production. Data available in the literature regarding EPS compositions have been gathered in Table 1, Table 2, Table 3, Table 4, Table 5, Table 6 and Table 7 (Table 1: Charophyta, Table 2: Chlorophyta, Table 3: Myozoa and cercozoa, Table 4: Haptophyta, Table 5: Ochrophyta, Table 6: Rhodophyta, Table 7: cyanobacteria), including, also, some newly discovered producing strains, for which no compositional analysis has been performed before. Additionally, compositional data have been used to construct a heat map (Figure 1), allowing a quick overview of similarities and differences between the main phyla. It is of note that the compositions reported by authors could strongly depend on culture conditions and downstream processes, but also on the analytical methodologies [27,28,29]. It is then not rare to observe a strong variability of published compositions for the same strain. This fact has been highlighted, for instance, in *Arthrospira platensis* EPSs by [29].

Despite a few exceptions, EPSs from microalgae and cyanobacteria are very complex heteropolymers, with a very high molecular mass, often found to be > 10^6^ Da. Their composition varies between 3 and 8 monosaccharides, except the one produced by *Gyrodinium impudicum* which appeared to be a sulfated galactan [30]. Additionally, they often contain uronic acids, and non-sugar groups, such as sulfate or methyl groups, on their backbone. Uronic acids will confer a negative charge to the polymer, that can influence biological and/or physico-chemical properties. The presence of non-sugar components is also of great importance because they can also influence these properties. For example, biological activities could be linked to the presence of sulfate groups, while methyl groups can induce large viscosity by hydrophobic interactions. Proteins are also often encountered in the samples studied, but are probably contaminants and not part of the EPS. As discussed later, there is not clear evidence on whether they could be covently bound to the EPS. Nevertheless, it is important to quantify them as, in some cases, it cannot be excluded that some properties of samples could be modified by their presence. Some studies are limited to neutral sugar analysis, and the quantification of uronic acids is often performed by a colorimetric assay. All neutral sugars, which usually enter in the composition of natural PSs, are found among these EPSs (xylose, glucose, galactose, mannose, rhamnose, arabinose, fucose), leading to very diverse compositions, in particular, among microalgae. In cyanobacteria, compositions seemed to be more conserved among evolution, with glucose generally found as the main or second monosaccharide, even if they also generally contain up to 6 or more monosaccharides [5,31].

When analyzed, the uronic acids are often identified as glucuronic and galacturonic acids (GlcA and GalA), sometimes at high levels, as in *Corynoplastis japonica*, *Neorhodella cyanea*, *Netrium interruptum*, *Netrium oblongum*, *Exanthemacrysis* sp., or *Phormidium corium* (674A), where they represent respectively 37, 35, 28, 29, 37, and 40% of the overall composition of the polymers [32,33,34]. In some cases, one of these uronic acids is even the main or second monosaccharide in EPS compositions, such as for *Closterium* sp. [35], *Staurastrum orbiculare* [36], *Amphora* sp. [37], *Anabaena spiroides* [38], *Aphanothece halophytica* [39], *Arthrospira platensis* [40], *Cyanospira capsulata* [41], *Fischerella maior* [42], *Corynoplastis japonica*, and *Neorhodella cyanea* [32]. In addition, some other uronic acids have been occasionally described, such as mannuronic acid, detected only in the EPSs of *Heterosigma akashiwo* [43]. The EPSs of *Oscillatoria* sp. and *Anabaena cylindrica* also have a non-identified uronic acid [44,45]. The occurrence of such acidic sugars on the EPS backbone is not scarce, as for compositions gathered in Table 1, Table 2, Table 3, Table 4, Table 5, Table 6 and Table 7, as at least 176 over 229 compositions present this specificity (~77% of the EPS compositions).

In some cases, and mainly for cyanobacteria, some osamines (GlcN and GalN) and their acetylated derivatives (GlcNAc and GalNAc) have also been detected. This is, for instance, the case for *Rhabdoderma rubrum* [45] and *Synechocystis aquatilis* [46], for which GlcN was found as the main or second monosaccharides, but also, to a lesser extent, for *Stauroneis* sp. and *Amphora* sp. [37], *Amphora salina* and *Triceratium dubium* [47], *Aulacoseira granulate* and *Microcystis aeruginosa* [38], *Thalassiosira* sp. [48], *Toypothrix tenuis* [49], *Calothrix* sp. and *Trichormus variabilis* [46], *Chroococcus minutus* and *submarinus* [45,50], *Gloeocapsa kuetzingigiana*, *Gloeocapsosis crepidinium*, *Plectonema* sp. and *Leptolyngbya* sp. [51], *Microcoleus vaginatus* and *Phormidium tenue* [31], *Synechocystis* sp. [52], or various *Anabaena* species [38,46,49]. The appearance of such animated monosaccharides is not really surprising in cyanobacteria as similarities can be found in the peptidoglycan layer of non-photosynthetic bacteria.

The non-sugar groups, such as sulfates and proteins, are not systematically analyzed in the available studies. Their presence or absence remains undetermined in about half of the compositions presented in Table 1, Table 2, Table 3, Table 4, Table 5, Table 6 and Table 7. As previously mentioned, this is detrimental as both can have an impact on biological activities. For EPSs in which sulfate groups have been quantified, it appears that this feature is widespread among diversity as, for the strains gathered in Table 1, Table 2, Table 3, Table 4, Table 5, Table 6 and Table 7, 78 EPSs out of 102 (more than 75%) show a sulfation pattern with, depending on the strains, some amounts being > 10%, as for the cyanobacteria *Arthrospira platensis* [53], *Nostoc* sp. [54], *Microcoleus vaginatus* [55], *Gloeothece* sp. [56] or *Johannesbaptistia pellucida*, *Chroococcus submarinus*, *Rhabdoderma rubrum*, *Aphanocapsa halophytia,* and *Phormidium battersii* [45], as well as for the microalgae *Amphora* sp. [57], *Gyrodinium impudicum* [30], *Cylindrotheca fusiformis* [28], *Porphyridium marinum* [13], *Porphyridium cruentum* and *Porphyridium aerugineum* [58], *Tetmemorus brebissonii* and *Pleurotaenium trabecula* [53], as well as the recently analyzed EPSs from *Timspurckia oligopyrenoides*, *Neorhodella cyanea*, *Chroodactylon ornatum*, *Chroothece richteriana*, *Bangiopsis subsimplex*, *Rhodospora sordida*, and *Rhodaphanes brevistipitata* with, for some of these latter strains, contents even greater than 20% [32]. Among these microalgae, it has been admitted for years that Rhodophyta produces sulfated polysaccharides. Until really recently [32], only a small number of EPSs from red microalgae had been studied (mostly *Porphyridium* and *Rhodella* strains) regarding their diversity. Interestingly, a few years ago, an EPS from *Flintiella sanguinaria* was found to be a 5.1% (*w*/*w*) methylated and 3.2% (*w*/*w*) acetylated polymer [59], with no sulfate groups (<0.6% *w*/*w*). However, in this recent study, 11 microalgae strains belonging to the proteorhodophytina phylum were studied for their ability to produce EPSs, and a preliminary approach was conducted on their structure. As for *F. sanguinaria*, three EPSs were found to contain a really low level of sulfate groups (*Erythrolobus coxiae*, 1.6%; *Erythrolobus madagascarensis*, 1.9%; and *Corynoplastis japonica*, 1.2%). Even if no quantification was performed on the *O*-methyl and *O*-acetyl groups, their presence is suspected in all EPSs from porphyridiophyceae and rhodellophyceae strains studied, i.e., *Erythrolobus coxiae*, *Erythrolobus madagascarensis*, *Timspurckia oligopyrenoides*, *Porphyridium sordidum*, *Neorhodella cyanea*, and *Corynoplastis japonica*. The presence of methyl groups was already described for other microalgae as it appears in 21 compositions in Table 1, Table 2, Table 3, Table 4, Table 5, Table 6 and Table 7, whereas acetyl groups were only identified in *Nostoc insulare* [60]. Nevertheless, the search for these patterns is rare in studies, and their occurrence could be much more frequent than currently described. Identification of the position of these methyl groups is even more rare, but it can be cited 3-*O*-Me-Xylose, 4-*O*-Me-Xylose, 2,3-di-*O*-Me-Rhamnose, and 2,3-di-*O*-Me-Fucose for *Rhodella grisea* [61], while di-*O*-Me-hexose, 4-*O*-Me-galactose [62], and 6-*O*-Me-mannose [63] have been detected in EPSs of *Porphyridium* sp. Additionally, 3-*O*-Me-Rhamnose has been identified in EPSs from *Arthrospira platensis* [64]. Earlier work from [58] on EPSs from *Porphyridium cruentum* has also shown that methyl groups can be found on uronic acids, as 2-*O*-Me-glucuronic acid was highlighted.

Pyruvate was only highlighted in the EPSs of *Chlamydomonas reinhardtii* [65], *Amphora rostrata* [66], and *Navicula subinflata* [67]. Proteins are often present at significant levels, but there is still no clear evidence for a linkage between the polysaccharide moiety and the protein fraction. Downstream processes may then strongly influence this protein level. Indeed, even with a well-conducted purification procedure, it seems nearly impossible to efficiently remove all proteins from samples, probably due to strong interactions with the polymer. Some proteases can be used to efficiently degrade these proteins [59], but additional EPS purification steps are needed afterwards to remove the enzymes.

A meta-analysis of 81 EPS compositions from Chlorophyta, Charophyta, Rhodophyta, Ochrophyta, and Haptophyta has been performed, intending to highlight some specificities related to the taxonomic affiliation of EPS producers [25]. Even if a great variability of compositions is recorded in the literature (even for the same strain) due to different production/purification/analysis methods, this study has allowed the observation of significant differences between phyla. As an example, EPSs from Charophyta present a level in fucose significantly greater than for EPSs from Chlorophyta and Rhodophyta (*p* < 0.001), as well as higher levels in uronic acids (*p* = 0.001). In the Chromista kingdom, significant differences were also found between Ochrophyta and Haptophyta, the first phyla being characterized by a high level in fucose, whereas the second one presents a significant level in arabinose. Finally, xylose is dominant in EPSs from Rhodophyta, whereas glucose is the main monosaccharide in EPSs from cyanobacteria [25]. When looking at the heat map proposed on Figure 1, the predominance of xylose as the main monosaccharide for Rhodophyta, and glucose for cyanobacteria can be clearly seen, whereas more diversity is observed for other phyla.

**Table 1:** Monosaccharide composition of EPSs produced by Charophyta.

Genera	Species	Rha	Gal	Ara	Glc	GlcA	GalA	Man	Fuc	Xyl	Rib	SO_4_^2−^	Proteins	Other Informations	References
*Closterium*	sp.														[35]
*Cosmarium*	sp.													Uronic acids	[33]
*Cosmarium*	sp.														[33]
*Hyalotheca*	*dissiliens*														[68]
*Klebsormidium*	*flaccidum* (749B)													Uronic acids	[34]
	*flaccidum* (748A)													Uronic acids	
	*flaccidum* (446C)													Uronic acids	
*Micrasterias*	*denticulata*														[69]
*Netrium*	*digitus*													Uronic acids	[33]
	*interruptum*													Uronic acids	
	*Interruptum UTEX 2509*														
	*oblongum*													Uronic acids	
*Penium*	*cylindrus*													Uronic acids	[33]
	*spirostriolatum*													Uronic acids	
	*margaritaceum*													Methyl groups	[70]
*Pleurotaenium*	*trabecula*													Uronic acids	[33]
*Spondylosium*	*panduriforme*														[71]
*Staurastrum*	*iversenii*														[72]
	*orbiculare*														[48]
*Stauroneis*	sp.													GlcN	[37]
*Tetmemorus*	*brebissonii*													Uronic acids	[33]

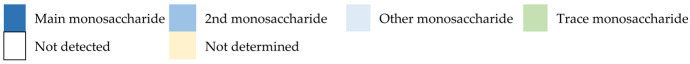

**Table 2:** Monosaccharide composition of EPSs produced by Chlorophyta.

Genera	Species	Rha	Gal	Ara	Glc	GlcA	GalA	Man	Fuc	Xyl	Rib	SO_4_^2−^	Proteins	Other Informations	References
*Ankistrodesmus*	*densus*														[73]
*Botryococcus*	*braunii*														[74]
	*braunii*													Methyl groups	[75]
	*braunii*													Uronic acids, methyl groups	[76]
*Bracteacoccus*	sp.													Uronic acids	[34]
*Chlamydomonas*	*agustae*														[77]
	*corrosa*														
	*humicola*														[78]
	*peterfii*														
	*reinhardtii*													Pyruvate	[65]
	*mexicana*													Uronic acids	[79]
	*sajao*													Uronic acids	
	*sajao*														[78]
	*stigmatophora*														[80]
	sp.														[81]
*Chlorella*	*autotrophica*														[82]
	*mirabilis* (678F)													Uronic acids	[34]
	*mirabilis* (7410G)													Uronic acids	
	*ellipsoidea*													Uronic acids	
	*pyrenoidosa*														[83]
	*vulgaris*														[81]
	*ellipsoidea*														
	sp.														
	sp.														[25]
*Chlorococcum*	sp.														[84]
*Coccomyxa*	sp.														[25]
*Desmococcus*	*olivaceus*													Methyl groups	[31]
*Dunaliella*	*bardawil*														[82]
	*tertiolecta*														[85]
	*salina*														[86]
	sp.														[25]
*Geminella*	*terricola*													Uronic acids	[34]
*Haematococcus*	*lacustris*														[87]
*Heterosigma*	*akashiwo*													ManA	[43]
*Oocystis*	sp.														[81]
*Palmella*	*mucosa*														
*Prasinococcus*	sp.														[25]
*Scenedesmus*	*quadricauda*														[88]
*Stichococcus*	*bacillaris* (772B, 747C)													Uronic acids	[34]
	*bacillaris* (774E, 677A)													Uronic acids	
*Tetraselmis*	*chui*														[25]
	*globosa*														
	*rubens*														
	sp.														

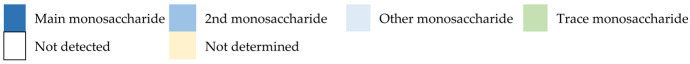

**Table 3:** Monosaccharide composition of EPSs produced by Myozoa and Cercozoa.

Genera	Species	Rha	Gal	Ara	Glc	GlcA	GalA	Man	Fuc	Xyl	Rib	SO_4_^2−^	Proteins	Other Informations	References
*Amphidinium*	*carterae*														[25]
*Cochlodinium*	*polykrikoides*														[89]
*Crypthecodinium*	*cohnii*														[90]
*Gyrodinium*	*impudicum*														[30]
*Bigelowiela*	*natans*														[25]

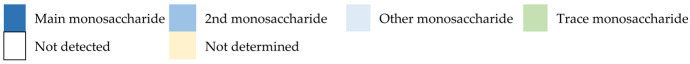

**Table 4:** Monosaccharide composition of EPSs produced by Haptophyta.

Genera	Species	Rha	Gal	Ara	Glc	GlcA	GalA	Man	Fuc	Xyl	Rib	SO_4_^2−^	Proteins	Other Informations	References
*Calyptrosphaera*	sp.														[25]
*Chrysotila*	*dentata*														[25]
	sp.														[25]
*Diacronema*	sp.														[25]
*Emiliania*	*huxleyi*														[25]
*Exanthemachrysis*	sp.														[25]
	sp.														[25]
*Hymenomonas*	*coronata*														[25]
*Isochrysis*	*braarudii*														[25]
*Ochrosphaera*	*verrucosa*														[25]
	sp.														[25]
*Pavlova*	*enorae*														[25]
	*gyrans*														[25]
	sp.														[25]
	sp.														[25]
	sp.														[25]
	sp.														[25]
*Prymnesium*	*parvum*														[25]
*Rebecca*	sp.														[25]
*Ruttnera*	*lamellosa*														[25]
PLY 431															[25]
RCC 3704															[25]

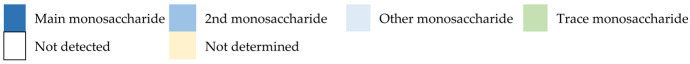

**Table 5:** Monosaccharide composition of EPSs produced by Ochrophyta.

Genera	Species	Rha	Gal	Ara	Glc	GlcA	GalA	Man	Fuc	Xyl	Rib	SO_4_^2−^	Proteins	Other informations	References
*Achnanthes*	*longipes*														[91]
*Amphora*	*coffeaeformis*														
	*holsatica*													Uronic acids	[92]
	*rostrata*													Pyruvate	[66]
	*salina*													GlcNAc	[47]
	sp.													GlcN, traces GlcNAc	[37]
*Asterionella*	*socialis*														[93]
*Aulacoseira*	*granulata*													GlcNAc	[38]
*Chaetoceros*	*affinis*														[94]
	*curvisetus*														[95]
	*decipiens*														[96]
	*decipiens*														[97]
*Coscinodiscus*	*nobilis*														[98]
	*radiatus*														[47]
*Cylindrotheca*	*closterium*													Uronic acids	[99]
	*fusiformis*													Uronic acids	[28]
*Cymbella*	*cistula*														[91]
	*mexicana*														
*Cyclotella*	*nana*														[93]
*Glossomastix*	sp.														[25]
	sp.														
	sp.														
*Melosira*	*nummuloides*													Uronic acids	[92]
*Navicula*	*directa*													Uronic acids	[92]
	*curvilineata*														[47]
	*incerta*														[93]
	*phyllepta*														[100]
	*salinarum*													Uronic acids	[99]
	*subinflata*													Uronic acids, pyruvate, methyl groups	[67]
*Nitzschia*	*angularis*													Other main sugar	[93]
	*closterium*														[85]
	*epithemoides*														[100]
	*frustulum*														[93]
*Pelagococcus*	sp.														[25]
*Phaeodactylum*	*tricornutum* CCMP632 ovoid													Methyl groups	[101]
	*tricornutum* CCMP632 fusiform													Methyl groups	
	*tricornutum*														[85]
*Phaeomonas*	sp.														[25]
*Pinnularia*	*viridis*													Uronic acids, methyl groups	[102]
*Thalassiosira*	*pseudonana*														[103]
	sp.													GlcNAc, GalNAc,	[48]
*Tribonema*	Sp.														[104]
*Triceratium*	*dubium*													GlcNAc	[47]

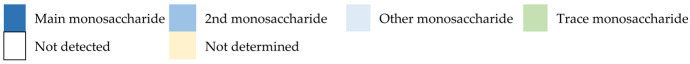

**Table 6:** Monosaccharide composition of EPSs produced by Rhodophyta.

Genera	Species	Rha	Gal	Ara	Glc	GlcA	GalA	Man	Fuc	Xyl	Rib	SO_4_^2−^	Proteins	Other Informations	References
*Dixionella*	*grisea*													Uronic acids, methyl groups	[11]
*Flintiella*	*sanguinaria*													Methyl and acetyl groups	[59]
*Porphyridium*	*aerugineum*													Methyl groups	[58]
	*cruentum*														[105]
	*cruentum*													Methyl groups	[58]
	*marinum*														[13]
	*purpureum*														[106]
	*purpureum*														[107]
	*sordidum*													Methyl groups	[107]
	*sordidum*														[32]
	sp.													Uronic acids, methyl groups	[62]
	sp.														[108]
*Timspurckia*	*oligopyrenoides*														[32]
*Erythrolobus*	*coxiae*														[32]
	*madagascarensis*														[32]
*Corynoplastis*	*japonica*														[32]
*Rhodella*	*grisea*													Methyl groups	[61]
	*maculata*														[106]
	*reticulata*														[109]
	*violacea*														[12]
*Neorhodella*	*cyanea*														[32]
*Chroodactylon*	*ornatum*														[32]
*Chroothece*	*richteriana*														[32]
*Bangiopsis*	*subsimplex*														[32]
*Rhodaphanes*	*brevistipitata*														[32]
*Rhodospora*	*sordida*														[32]

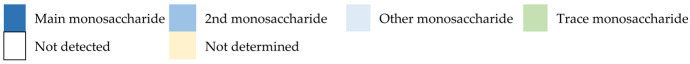

**Table 7:** Monosaccharide composition of EPSs produced by Cyanobacteria.

Genera	Species	Rha	Gal	Ara	Glc	GlcA	GalA	Man	Fuc	Xyl	Rib	SO_4_^2−^	Proteins	Other Informations	References
*Anabaena*	*augstmalis*													GalN	[46]
	*cylindrica*													Other uronic acid	[44]
	*flos-aquae*														[81]
	sp.													Uronic acids, pyruvate	[110]
	*spiroides*													GlcNAc, GalNAc	[38]
	*sphaerica*														[49]
	*torulosa*													GlcN	
*Anacystis*	*nidulans*														[111]
*Aphanocapsa*	*halophytia*														[112]
*Aphanothece*	*halophytica*														[39]
*Arthrospira*	*maxima*														[113]
	*platensis*														[114]
	*platensis*														[53]
	*platensis*													Methyl groups	[40]
*Calothrix*	*pulvinata*													Uronic acids	[34]
	sp.													GlcN	[46]
*Chlorogloeopsis*	sp.														[49]
*Chroococcus*	*minutus*													GlcN, methyl groups	[50]
	*submarinus*													GlcN	[45]
*Cyanospira*	*capsulata*														[41]
*Cyanothece*	sp.														[115]
	sp.														[116]
*Fischerella*	*maior*														[42]
	*muscicola*														[49]
*Geitlerinema*	sp.														[45]
*Gloeocapsa*	*gelatinosa*														[117]
	*kuetzingigiana*													GalN, traces GlcN	[51]
	sp.														[118]
*Gloeocapsosis*	*crepidinum*													GlcN, GalN	[51]
*Gloeothece*	PCC6909														[119]
	sp.													Methyl groups	[56]
*Johannesbaptistia*	*pellucida*														[45]
*Leptolyngbya*	*foveolarum*													Uronic acids	[34]
	*tenuis*													Uronic acids	
	sp.													GalN, traces GlcN	[51]
*Lyngbya*	*conferviodes*														[120]
*Mastidocladus*	*laminosus*														[121]
*Microcoleus*	*vaginatus*														[55]
	*vaginatus*													GlcNAc, methyl groups	[31]
*Microcystis*	*aeruginosa*													GlcNAc, GalNAc	[38]
	*aeruginosa*														[122]
	*aeruginosa flos-aquae*														
	*viridis*														
*Nostoc*	*calcicola*														[78]
	*carneum*														[116]
	*commune*														[123]
	*commune*														[124]
	*flagelliforme*														[123]
	sp.														[54]
	sp.														[81]
	sp.													Methyl groups	[125]
	*insulare*													Methyl groups	[60]
	*insulare*														[50]
	*muscoru*														[118]
	*entophytum*														
	sp.														[46]
	*verrucosum*														[126]
*Oscillatoria*	*amphibia*														[120]
	*corallinae*														[120]
	*planktothrix* FP1														[127]
	sp.														[49]
	sp.														[116]
*Phormidium*	*autumnale*														[46]
	*battersii*														[45]
	*ambiguum*													Uronic acids	[34]
	*corium* (442D, 746B)													Uronic acids	
	*corium* (444A)													Uronic acids	
	*corium* (674A)													Uronic acids	
	*corium* (743D)													Uronic acids	
	*ectocarpi* (K5)														[120]
	*ectocarpi* (ME3)														
	*ectocarpi* (N182, C86)														
	*joveolarum* (C52)														
	*joveolarum* (MEU)														
	*minutum* (D5)														
	*minutum* (NB5)														
	*minutum* (RT6)														
	sp. (CCAP1464)														
	sp. (PNG91, 90-14/1)														
	sp.														[49]
	*tenue*													GlcNAc	[31]
	*tenue*													GlcNAc	[125]
	*golenkinianum*														[45]
	sp.													GlcN, traces GalN	[51]
*Rhabdoderma*	*rubrum*													GlcN	[45]
	*javanicum*													Methyl groups	[31]
	*javanicum*														[125]
*Synechococcus*	sp.														[128]
	sp.														[25]
*Synechocystis*	*aquatilis*													GlcN	[46]
	sp.														[118]
	sp. (PCC6803)													GlcN, GalN	[52]
	sp. (PCC6714)													GlcN, GalN	
*Tolypothrix*	*tenuis*													GlcN	[49]
*Trichormus*	*variabilis* (VRUC162)													GalN	[46]
	*variabilis* (VRUC168)													GlcN	

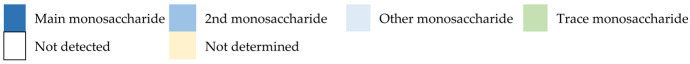

If the number of published EPS compositions have increased over recent years, there is still a lack of complete structures’ characterization. This could be explained by the high complexity of microalgae and cyanobacteria polymers, related to the numerous different monosaccharides included, the almost systematic presence of non-sugar substituents, and the apparent lack of repeating units. To date, only partial information has been published, and on a very limited number of EPSs produced by Chlorophyta (*Chlamydomonas augustae*, *Chlamydomonas corrosa*, *Ankistrodesmus densus*), Rhodophyta (*Porphyridium* sp., *Porphyridium cruentum*, *Porphyridium aeruginosum*, *Rhodella reticulata*), or cyanobacteria (*Cyanothece* sp., *Cyanospira capsulata*, *Mastidocladus laminosus*, *Oscillatoria planktothrix*, *Nostoc commune* and *Nostoc insulare*). These incomplete structures have been reported in Table 8. This lack of complete structures is unfortunate regarding the industrial valorization of EPSs, mainly because of the impossibility to establish a clear relationship between structures and biological activities and/or physico-chemical properties.

## 3. Culture Strategies for EPS Production and Impact on Composition

Concerning strategies enhancing EPS accumulation by microalgae and cyanobacteria, it is not obviously possible to define general trends as many comportments can be observed depending on species. This feature could probably be linked to the fact that microalgae belong to a polyphyletic group. Moreover, it is often difficult to determine the most accurate parameter for a strain only on a literature basis, as authors generally indicate only the final EPS concentration in the culture medium, without always mentioning the amount of biomass reached. This seems unfortunate as cellular productivities (the amount of EPSs per gram of biomass or per cell) appear to be a better way to evaluate process efficiency. One of the levers of optimization might, in the years to come, go through a better understanding of the metabolic pathways involved. Currently, if some piecemeal information is available for cyanobacteria [5,133] or red microalgae (for which floridoside has been identified as a precursor for PS synthesis in *Porphyridium*, [11]), these data remain incomplete. As an example, floridoside is the precursor of both EPSs and starch for this strain, and the mechanism by which carbon flux is directed towards one or the other PS is still unknown [134,135]. Actually, the optimization of production therefore implies a systematic study of each strain, in order to define, case by case, the most appropriate operating parameters.

Studying the EPS production by a microalgae or cyanobacteria strain requires, first, an efficient method to detect and quantify the polymer release. The detection of exopolysaccharide producers is not always coarse as EPSs can only be synthesized under specific culture conditions. Moreover, a negative result in a specific culture condition is not sufficient to conclude that the strain is a non-producing one. The easiest method for detection could be by assaying sugars in the culture supernatant, using classical sugar quantifications, such as the Dubois or Monsigny methods [136,137]. However, attention must be paid to possible interferences of the culture medium compounds with the assay reagents. It could then be necessary to remove the culture medium (by dialysis, for instance) prior to the assay. Moreover, these methods are not suitable for BPSs which need to be extracted before quantification. For EPSs that exhibit viscosity in solution, this behavior can be an indirect way to evaluate the release of polymers, as shown by [138], who compared the viscosity of nearly 800 microalgae and cyanobacteria culture supernatants with those of culture media alone. In the same way, a tool has been developed and successfully applied to the detection of viscous algal polysaccharides [139], even with really low variations in viscosity.

After the identification of a producing strain, the parameters potentially having an impact on the quantity of released EPSs should be screened. They are mainly of an operating nature and can be assimilated to stresses for the cells. Indeed, the availability of macro and micronutrients (and any condition of deficiency associated), salinity, temperature, or irradiance appear to be the key parameters to consider in order to improve the EPS production yields. Actually, as the metabolic pathways for EPS accumulation are not resolved, it is not possible to predict for one strain which parameter will have an impact and could be the most suitable. Thus, each author will test one or several parameters and observe an enhancement or not. Some published information about operating conditions and their impact on production yields for some microalgae and cyanobacteria strains have been gathered in Table 9. If nitrogen limitation will appear as a widespread way to induce EPS accumulation, it is, nowadays, not possible to conclude if it is the best parameter or not, as it could be due to the fact that it is the most studied parameter.

### 3.1. Nutrient Limitations

The nutrient limitation is the most common strategy to induce the accumulation of the compounds of interest and/or of secondary metabolites, such as pigments, PUFAs, and PSs.

The production of EPSs can be induced by the deficiency and/or the limitation of available nitrogen in the culture media, as indicated in Table 9, related to many studies conducted on different microorganisms such as microalgae similar to *Botryococcus braunii* [140], *Chlamydomonas* [141], *Chlorella* sp. [142], *Flintiella sanguinaria* [59], *Porphyridium* [13,32,143], *Rhodella* [12,144,145], *Tetraselmis gracilis* [146], *Corynoplastis japonica*, *Neorhodella cyanea*, *Erythrolobus coxiae*, *Erythrolobus madagascarensis*, or *Timspurckia oligopyrenoides* [32], but also for the cyanobacteria *Anabaena* sp. [147], *Anacystis nidulans* [111], *Cyanothece* [115,148,149], *Nostoc calcicola* [78], or *Spirulina* sp. [49], and diatoms *Thalassiosira Pseudonana* [103] or *Cylindrotheca closterium* [150]. Nevertheless, this same limitation in nitrogen has no effect on the production of EPSs in *Phormidium* [151,152], *Synechocystis* [52], or *Scenedesmus* [153]. For strains producing both soluble and bound polymers, nitrogen starvation can impact the repartition between both fractions, increasing the RPS amount, as shown for *Porphyridium* sp. [154] or *Rhodella reticulata* [144].

If the mechanism by which the nitrogen amount promotes EPS synthesis remains unclear, it has been proposed that a high C/N ratio may impact the carbon metabolism [155,156]. This hypothesis is supported by the fact that many studies show that the supplementation of CO_2_ during the culture affects the growth and production of various metabolites by the microalgae, including the EPSs. For example, an absence of CO_2_ supplementation in the culture leads to a decrease in EPS production for *Phormidium* sp. and *Anabaena torulosa* (9 mg L^−1^ instead of 22 and 56 mg L^−1^, respectively) [49]. Additionally, cultures of *Porphyridium* supplemented with 2.5% of CO_2_ were able to recover 1.4 g L^−1^ of EPSs in the supernatant, against 0.7 g L^−1^ without CO_2_ addition [157]. These findings are also comforted by the fact that the glyoxylate addition in the culture medium (a stimulator of carbon metabolism by increasing the CO_2_ fixation rate and inhibiting photorespiration) enhanced EPS synthesis by *Anabaena cylindrical* [158], as well as for *Chlorella pyrenoidosa* [159]. Furthermore, the EPS production by *C. capsulata* increased by 43% after a short exposure to glyoxylate [160]. However, the same nitrogen deprivation is also known to allow the accumulation of lipids or storage polysaccharides for some microalgae strains. It remains, then, obviously unknown the reason why the microalgae metabolism is oriented toward lipids or EPS synthesis depending on the species.

Some studies have also shown that EPS production can be influenced by the nature of the nitrogen source, such as for *Anabaena cylindrica* [44], or for *Botryococcus braunii* [135], while it had no effect on *Anabaena flos-aquae*, cultivated using KNO_3_ and NH_4_Cl [161]. However, before concluding on different final EPS concentrations, attention has to be paid to the potential different biomass productivities that can be observed varying the nitrogen source. For the diazotrophic cyanobacterium *Anabaena* sp., supplementation of the culture medium with nitrogen in the N_2_ form leads to a significant increase in EPS production compared with nitrates or ammonium sources (17, 2 and 2.7 g L^−1^, respectively, [147]). The same conclusion has been obtained for *Synechococcus* [162] and several *Anabaena* strains [44,49], while it was not the case for *Nostoc* sp. [156] or *Phormidium laminosum* [163]. The mechanism by which N_2_ contributes to EPS synthesis remains unknown, but as the availability of resulting nitrogen could be low, the hypothesis is that it could be linked to a N limitation and a high C/N ratio, as previously described.

Even if it is the most studied, nitrogen is not the sole nutrient for which starvation may induce EPS release. Phosphorus and sulfur depletions can also promote EPS production, even if studies regarding sulfur are scarce (Table 9). A positive impact of phosphorus deprivation has been shown for the cyanobacteria *Spirulina* sp. [49] and *Cyanothece* [115], microalgae *Chlorella* sp. [164] and *Porphyridium marinum* [13], and diatoms *Cylindrotheca closterium* [150,165], *Thalassiosira pseudonana* [165], *Chaetoceros affinis* [94], and *Cylindrotheca fusiformis* [28]. Conversely, phosphorus limitation had no effect on the production of EPSs by *Phormidium* [49,151] and *Anabaena torulosa* [49], and it may even be reduced for *Anabaena cylindrica* [44]. For the diatom *Achnanthes brevipes*, EPS synthesis is increased by phosphate starvation, whereas nitrogen depletion had no effect. Moreover, combined nitrogen and phosphorus deprivations were shown to have a negative effect, as they induced the accumulation of intracellular carbohydrates [166].

For sulfur starvation, no modification of production yields was noticed for *Rhodella reticulata*, but it has been demonstrated that the degree of the sulfation of polymers was reduced by half [144,154]. In addition, the degree of the sulfation of EPSs produced by *Porphyridium cruentum* was improved with a 104 mM concentration in MgSO_4_ instead of 21 mM [105].

The micronutrients may also have a role in the production of EPSs (Table 9). Indeed, this last is improved in the deficiency of the calcium cation for *Phormidium* [151]. For *Cyanospira capsulata*, a 17% increase in the production of EPSs was observed in the condition of a magnesium or calcium deficiency [167]. Conversely, the productivity was found lower for *Cyanothece* in calcium (379 mg L^−1^) or magnesium (238 mg L^−1^) deficiency conditions, as compared with the control (500 mg L^−1^) [115].

In addition to the activation and/or the improvement of productivity in EPSs, the nutrient deficiency may also affect the composition of monosaccharides. Glucose is the main monosaccharide of the EPSs of *Cylindrotheca closterium*, *Thalassiosira pseudonana*, and *Skeletonema costatum* during the exponential growth phase in a replete medium [165]. In case of phosphorus depletion, its content decreased for *Cylindrotheca closterium*, while for *Thalassiosira pseudonana*, the fucose amount was significantly reduced (from a 30% molar ratio in the EPSs to 5%) [165]. When grown in a replete medium, *Cylindrotheca closterium*, *Navicula perminta*, and *Amphora exigua* produced complex EPSs containing Rha, Fuc, Xyl, Man, Gal, Glc, and uronic acids [168]. An analysis of the monosaccharides composition of the EPSs produced by *Cylindrotheca fusiformis* has shown that a nitrogen deficiency led to a specific decrease of the content in Gal, Man, and Xyl [28], and an increase in the level of Fuc and Rha. For the P deficiency condition, the Glc and Man amounts declined, but those of Rha and Gal increased. Moreover, a significant increase (50%) of the uronic acids level in the polysaccharide was observed in the P starvation condition, whereas the sulfation degree decreased by half in the N limitation condition [28]. However, due to a lack of knowledge on the metabolic pathways involved, it is not possible to understand by which mechanisms these compositions are impaired.

Depending on the strain, EPS production can also be observed throughout the growth phase and then be improved as soon as the entry into the stationary phase occurs, as for the cyanobacteria *Aphanocapsa halophytica* [112], *Cyanospira capsulata* [169], *Nostoc insulare* [60], *Chroococcus minutus* [50], *Anacystis nidulans* [111], *Synechocystis* [52], *Anabaena flos-aquae* [81,156], *Anabaena cylindrical* [44] et *Synechococcus* sp. [128], and for the microalgae *Botryococcus braunii* [140,170] and *Heterosigma akashiwo* [43]. For those strains, the nutrient deficiency strategy can also be successfully used to improve the EPS production. However, for others, the stationary phase did not lead to an increase in EPS release. As an example, the EPS production by *Chaetoceros affinis* is even more important during the exponential growth phase, comparing to its production during the stationary phase [171]. This is also the case for *Arthrospira platensis* [40], or *Nostoc* strains [172,173]. For these strains, other modulations of operating conditions could be preferred for the stimulation of EPS production.

### 3.2. Other Stresses

Apart from nutrient depletion, alternatives exist to induce the production of EPS by microalgae and cyanobacteria. They are based on the environmental parameters such as the salinity, irradiance, and temperature of the culture medium.

A high salinity strategy can be applied to improve EPS synthesis by some strains, but is limited to halophilic or at least halotolerant microalgae and cyanobacteria. One of the advantages is that this high salinity could help control bacterial contamination when cultures are conducted in open ponds. As an example, for a 5M NaCl concentration, the EPS production by *Dunaliella salina* is 17 times more important than for 0.5 M (944 mg L^−1^ and 56 mg L^−1^, respectively) [174]. A similar phenomenon has been observed for *Microcoleus vaginatus* [31,175], *Scytonema javanicum*, *Phormidium*, *Nostoc* sp., and *Desmococcus olivaceus* [31], *Spirulina* sp. [49], and *Scenedesmus* [153]. On the contrary, the EPS production by *Aphanocapsa halophyta* is strongly reduced beyond 4% NaCl and is avoided at 10% [112]. For *Anabaena* sp. the production was found to decrease from 13.5 to 9.2 and 3.9 g L^−1^ for NaCl concentrations of 0, 0.85, and 510 mM, respectively [147]. For *Synechocystis*, results are contradictory as [176] described a positive effect of salinity, whereas for [52], an increase in NaCl concentration up to 0.5 M failed to improve the EPS production, as for *Cyanothece* sp. for NaCl concentrations up to 2 M [115]. The impact of salinity on the synthesis of EPSs is, therefore, once more strain-dependent. For *Nitzschia frustulum*, strong variations in EPS levels were observed with the NaCl concentration, without having an impact on cell growth. However, the study failed to establish a linear relationship between the amount of EPSs produced and the salinity of the medium [93]. The EPS composition was affected, with levels of Man, Gal, and Fuc reduced by a factor of 3, 4, and 6 when the strain was grown with 20.8 g L^−1^ of NaCl instead of 1.3 g L^−1^. On the other hand, the levels of Rha and Xyl increased by a factor of 2 and 7. The study of [177] on *P. purpureum* has shown that EPS production increased by 32 g L^−1^, as compared to 18 and 50 g L^−1^. However, the EPS composition was not found significantly different, except for a change in the sulfation pattern, with sulfate groups at the *O*-6 position on glucose residues at low salinity, whereas high salinity induced sulfation on the *O*-4 position.

The release of EPSs can also be improved by a continuous supply of light [138] and high irradiances [147] that can, in some cases, be assimilated as a stress for the cells. To support this hypothesis, [178] have shown that EPS synthesis occurred simultaneously with those of photoprotective pigments during the application of UV-B radiations. The effect of irradiance has been studied in *Rhodella violacea* [12], *Porphyridium marinum* [13], or *Flintiella sanguinaria* [59], showing that the best EPS productivities were reached for the irradiance of saturation, just before the one inducing the photoinhibition. The EPS yield produced by the cyanobacteria *Nostoc* sp or *Microcoleus vaginatus* increased with light intensity, but no differences in the monosaccharide composition were noticed [54,55]. For *Arthrospira platensis*, cultivation under 800 µmol photons m^−2^ s^−1^ induced the production of EPSs by up to 25–30% of their dry weight (against about 10–15% DW at 100 µmol photons m^−2^ s^−1^). Nevertheless, this high light condition has also been shown to induce glycogen accumulation, and to modify EPS composition [29]. For high incident light fluxes, the amount of Glc in the polymer was found to increase up to 77% (% molar ratio in the EPS), detrimentally to the other neutral sugars and uronic acids amounts. Moreover, the sulfation level was also found to be reduced.

In some cases, the light spectrum also affects the photosynthetic activity and thus the EPS production. Indeed, [86] have compared the EPS production by *Porphyridium cruentum* while using red light (600–700 nm), blue light (400–500 nm), or white light. For low irradiances (20–40 µmol photons m^2^ s^−1^), these specific wavelengths were found to improve the accumulation. Additionally, red light was also found to stimulate polysaccharide production (both capsular and soluble fractions) in the cyanobacteria *Nostoc flagelliforme* [179]. On the other hand, the long term (5 months), continuous cultivation of *Arthrospira platensis* under red light (135 µmol photons m^2^ s^−1^) has shown no differences in the growth rate and EPS production, as compared to white light [180].

Finally, temperature can also be a parameter allowing the inducing of EPS synthesis. *Nostoc* sp. produces more EPSs when it is grown at 30 °C rather than 35 °C, in the presence of NO_3_^−^ ions [156]. This phenomenon is not observed when N_2_ is the sole source of nitrogen. However, this result has to be taken with care as the growth rate of the strain is increased at 30 °C, which can explain the greater EPS concentration observed. For *Botryococcus braunii*, the best EPS production could be observed at 28 °C, with a decrease for a lower (20–23 °C) or greater temperature (32 °C), and is zero at 33 °C [181]. The lack of production of EPSs at 33 °C can thus be explained by a growth inhibition. On the other hand, with the productivity of the biomass being similar at 20, 23, and 28 °C, it is possible to conclude that temperature has an impact on the production of EPSs for this strain. An increase in the EPS production by a factor of 5 was also observed for *Anabaena* sp. when the culture temperature shifted from 30–35 °C to 40–45 °C [147].

Finally, in a recent study, [182] have studied the effect of a static magnetic field on *Limnospira indica* PCC8005 (formerly known as *Arthrospira indica* PCC8005) EPS production and composition. If static magnetic fields have an impact on the growth rate, pigment content, and intracellular polysaccharide accumulation, it seems that the EPS production yield is not increased. However, the EPS composition was found modified, with a greater content in glucuronic acid, fucose, arabinose, and rhamnose, while the glucose and xylose contents were decreased.

**Table 9:** Already tested operating conditions and their impact on production yields for some microalgae and cyanobacteria strains.

		Growth Phase		Starvation			Other Stresses		
	Strain	Exponential	Stationary	N	P	Ca^2+^ or Mg^2+^	High Salinity	High Light	References
c	*Anabaena cylindrica*	+	++		−				[44]
c	*Anabaena flos-aquae*	+	++						[81,161]
c	*Anabaena* sp.		+	+			−		[147]
c	*Anabaena torulosa*				−				[49]
c	*Anacystis nidulans*	+	++	+					[111]
c	*Aphanocapsa halophyta*						−		[112]
c	*Aphanocapsa halophytica*	+	++						[112]
c	*Arthrospira platensis*	+	−	−			−	+	[29,40]
c	*Chroococcus minutus*	+	++						[50]
c	*Cyanospira capsulata*	+	++			+			[162]
c	*Cyanothece*		+	+	+	−	−		[115,148,149,169]
c	*Microcoleus vaginatus*						+	+	[31,54,55,175]
c	*Nostoc calcicola*		+	+					[78]
c	*Nostoc insulare*	+	++						[60]
c	*Nostoc* sp.	+	−				+	+	[31,54,55,167,173]
c	*Phormidium*			−	−	+	+		[31,49,151,152]
c	*Scytonema javanicum*						+		[31]
c	*Spirulina* sp.		+	+	+		+		[49]
c	*Synechococcus* sp.	+	++						[128]
c	*Synechocystis*	+	++	−			+/−		[52,176]
d	*Achnanthes brevipes*			−	+				[166]
d	*Chaetoceros affinis*	+	−		+				[94]
d	*Cylindrotheca closterium*		+	+	+				[150,165]
d	*Cylindrotheca fusiformis*		+		+				[28]
d	*Nitzschia frustulum*						+		[93]
d	*Thalassiosira Pseudonana*		+	+					[103,165]
m	*Botryococcus braunii*	+	++	+					[140,170]
m	*Chlamydomonas*		+	+					[141]
m	*Chlorella* sp.		+	+	+				[142,164]
m	*Desmococcus olivaceus*						+		[31]
m	*Dunaliella salina*						+		[174]
m	*Flintiella sanguinaria*		+	+				+	[59]
m	*Heterosigma akashiwo*	+	++						[43]
m	*Porphyridium aeruginum*		+	+					[143]
m	*Porphyridium marinum*		+	+	+			+	[13]
m	*Rhodella grisea*		+	+					[144]
m	*Rhodella reticulata*		+	+					[145]
m	*Rhodella violacea*		+	+				+	[12]
m	*Scenedesmus*			−			+		[153]
m	*Tetraselmis gracilis*		+	+					[146]

c: cyanobacteria, d: diatom, m: microalgae; +: greater production yield, − lower production yield.

### 3.3. Culture Strategies

Obviously, any of the existing culture systems (open ponds or photobioreactors, with natural or artificial light) may be used for EPS production by microalgae or cyanobacteria, as soon as the system is appropriate in supporting the growth of the microorganism with significant biomass productivity and within a reasonable time. However, attention has to be paid to the factor chosen to promote the synthesis, as all systems could not be suitable. As an example, an artificial lighting system could be preferred if a high light stress is required, as the control of the production process will be easier. The mixing system should also be carefully chosen, as the viscosity of the medium could drastically increase during production. An inappropriate mixing will then decrease the cells’ accessibility to light and nutrients, as well as gas transfers. Parameters to consider while the choosing production system will then be based on the cell physiology (growth rate, resistance to shear stress, etc.), as well as the efficiency of the production process regarding biomass productivity (gbiomass L^−1^ h^−1^), cellular EPS productivity (gEPS gbiomass^−1^), and global process productivity (gEPS L^−1^ h^−1^).

For strains for which EPS production is induced (or at least improved) by a condition of nutrient depletion, the main drawback is that a low rate of growth and a possible decline of biomass will be encountered in these conditions [183]. Indeed, the synthesis of proteins is impaired when the cells are placed in a state of deficiency [184]. A compromise between cell growth and the production of target molecules is therefore necessary. Usually, the production of compounds of interest is then carried out in a batch mode. After a growth phase in a replete medium, the production of EPS will start as soon as the conditions of depletion are fulfilled (entry into stationary phase). In some cases, a semi-continuous strategy can also be used in order to improve the process productivity. This strategy has been used recently by [13] on the red microalga *Porphyridium marinum*, leading to a process productivity of 0.031 gEPS h^−1^ instead of 0.020 gEPS h^−1^ in batch mode. However, it is viable only for the species which show a significant increase in the production of EPS in a nutrient starvation condition.

An easy way to control the nature of nutrient deficiency in a batch mode culture is to adjust the initial N/P ratio of the medium. The impact of the ratio of N/P on the production of biomass and total PS has been studied in *Porphyridium cruentum*. Ratios between 35 and 50, corresponding to a limitation in phosphate, have improved the production of biomass, while a ratio of N/P < 4.9, corresponding to a nitrogen limitation, improved the production of PS by two [185]. Accordingly, N/P ratios around four allowed a significant improvement in EPS production for *Rhodella violacea* [12], *Flintiella sanguinaria* [59], and *Porphyridium marinum* [13]. In this latter case, the EPS synthesis was found to be greater for N depletion than for P deprivation (N/P ratio of 3.97 and 24.5), with specific productivities of 0.0622 mg 10^−6^ cells and 0.0503 mg 10^−6^ cells, respectively. However, it could not be always the case, depending on the species studied.

For some strains, a nitrogen limiting condition (and not complete deprivation) could be sufficient to improve EPS production, as shown for several *Cyanothece* strains [115,148], and then allowing a continuous mode strategy. In that case, and after a batch phase to reach the targeted biomass concentration, continuous cultivation could be conducted with a low level of nitrogen in the medium feed, allowing both growth and EPS production to be supported for a longer time.

For other induction methods, a two-step culture mode could be needed, as the parameter allowing the induction of production could be inconsistent with the optimal growth parameters. As an example, high incident light fluxes can induce photoinhibition and lead to cellular death. After a growth stage, when the biomass concentration has increased, the same incident light flux could be bearable for cells due to the shading of cultures. The light stress can then be applied. Another example is the high salinity that may inhibit growth, without leading to cellular mortality. In that case, the stress should be applied after the growth phase as a second step.

## 4. Processes for Extraction and Purification

**Figure 2:**
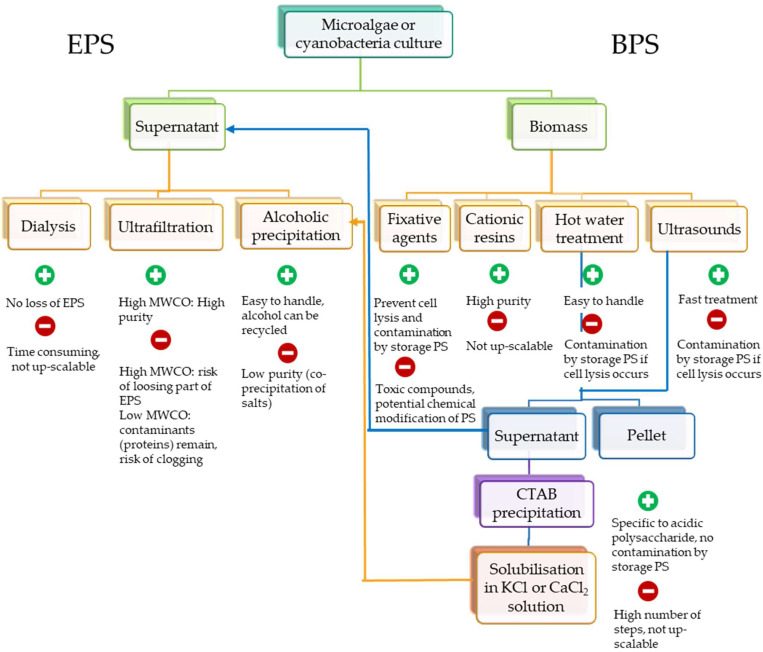
Overview of methods that can be applied to recover released EPSs or BPSs, main advantages, and drawbacks.

After growth of the microalgae or cyanobacteria in the appropriate conditions for EPS synthesis, it is necessary to implement some methods to recover the molecules. Some compromise has then to be done between the purity of the polymer, and the cost of the processes for extraction and purification. Different downstream processes will be required to recover RPSs or BPSs. Figure 2 provides an overview of methods that can be applied, highlighting their main advantages and drawbacks.

### 4.1. Released EPSs

The soluble EPSs in the culture medium are the easiest to recover. As a first step, they can be separated from the biomass by centrifugation, or membrane filtration [57,185,186,187]. Depending on the use of the EPSs, a purification step should be necessary to remove non-sugar compounds such as proteins, pigments, and salts. Different methods exist, each of them having advantages and drawbacks for scale-up at an industrial level. These are dialysis, tangential ultrafiltration, or selective alcoholic precipitation [81,188,189,190]. Indeed, the soluble fraction may be precipitated by alcohols of varying polarities, such as methanol, ethanol, or isopropanol [81,90,130,147,181,190,191]. Apart from the polarity, the temperature has to be controlled as the precipitation will be enhanced by cold temperatures. In order to reduce the volume of alcohol to be used (2–3 volumes of alcohol per volume of PS solution), a concentration step could be required, by filtration or concentration under a vacuum. Of course, this additional step will increase the cost of post-culture processes. Alcoholic precipitation is widely used at an industrial level to purify PSs from bacteria or higher plants, and the possibility of alcohol recycling makes this process eco-friendly. The main drawback regarding its use for EPSs from microalgae and cyanobacteria is the low purity obtained by this method, due to the co-precipitation of salts from the culture medium [190]. In this case, tangential ultrafiltration and dialysis could be used as alternatives to alcoholic precipitation. A comparative study between the techniques of alcoholic precipitation, dialysis, and tangential ultrafiltration was conducted by [190]. The results have shown that tangential ultrafiltration with a 300 kDa molecular weight cut-off (MWCO) polyethersulfone membrane was the most effective method. The same conclusion was found by [186]. These authors compared three techniques of concentrations/precipitations (ethanol precipitation, diafiltration in cell Amicon, and tangential ultrafiltration) in order to recover the EPSs produced by the diatom *Amphora* sp. The results show that the filtration techniques are the most effective to isolate the EPSs from a culture medium rich in salts. Other authors have used tangential filtration (with a membrane of 10 kDa MWCO) to concentrate the EPSs from *Nostoc commune*, before a step of alcoholic precipitation [124]. Additionally, [187] have developed a pilot-scale extraction and purification process for EPSs from *Chaetoceros muelleri*, *Chlorella pyrenoidosa*, *Spirulina platensis*, *Haematococcus pluvialis*, *Nostoc commune*, and *Nostoc sphaeroide*, with a microfiltration process (polypropylene membrane) to remove biomass, followed by ultrafiltration with a 5 kDa polyethersulfone membrane for purification and concentration.

The efficiency of membrane filtration systems for EPS purification is strongly dependent on the viscosity and concentration of the EPSs, the pore size distribution, and the flow rate and transmembrane pressure. Concerning pore size (or MWCO), a compromise has to be made between a high MWCO, which presents the risk of losing part of the EPS, but for which the purity will be great (as almost all proteins will be eliminated in the filtrate) while maintaining a reasonable flow rate, and a low MWCO, for which the flow rate will be significantly lower and allows the recovery of almost all of the sample, but probably with contaminating proteins. Many authors describe disadvantages related to the use of tangential ultrafiltration as a means of the extraction and purification of PSs. Among them, the phenomenon of clogging the membranes related to the viscosity of the EPSs is the most frequent [57,188,192]. The fouling of membranes resulted, therefore, in reducing all the flow performances and increasing the operating prices [57,188]. In addition, a large quantity of water is necessary to isolate, purify, and concentrate the EPSs by ultrafiltration [187]. However, in the light of recent advances in the area of membrane separation processes, the filtration could be used to extract and purify the EPSs from microalgae and cyanobacteria. The authors of [193] have recently published a review on the development of nanocomposite membranes to prevent clogging. As indicated by the authors, many nanocomposite membranes composed of nanoparticles on the polymer membrane (SiO_2_, TiO_2_, etc.) have been manufactured to improve the properties of anti-clogging, hydrophobicity, and self-cleaning. Moreover, the use of inorganic and organic membranes, such as AZT (Aluminum/Zirconium/Titanium Oxide) and polyacrylonitrile, could help control the clogging phenomenon while harvesting *Arthrospira platensis* [194,195].

At the present time, the cheapest industrial extraction remains without doubt the alcoholic precipitation. However, the development of membrane processes is a good alternative for the desalting and concentration of the EPS solutions, even if it constitutes a very limiting step because of the high viscosities of the solutions and the high cost of the membranes.

### 4.2. Cell-Bound PSs

For the specific case of PSs that remain more or less tightly bound to the cells, specific methods have to be used to selectively recover this fraction. Consequently, a universal method to release these PSs does not exist, and different methods can be used depending on the strain, such as hot water treatments, ethylene diamine tetracetic acid (EDTA), sodium hydroxide (NaOH), sonication, and so on [19,196,197,198]. Even if these methods are classically used to extract polysaccharides from seaweeds for which they have proven their efficiency, attention has to be paid to the treatment intensity in order to prevent the degradation of polysaccharides. Indeed, sonication or long and hot water treatments can be used specifically to decrease the molar mass of polysaccharides [199,200]. Mild conditions are thus recommended for the extraction of BPSs.

Regarding hot water treatment, the temperature (30 °C to 100 °C), the biomass concentration, the pH, and the duration of treatment (generally between 1 to 5 h) are the key parameters to control the release of PSs. A centrifugation or filtration step is, therefore, necessary to remove the cells, followed by the alcoholic precipitation or ultrafiltration of the supernatant containing the EPSs, as previously described. However, if cellular lysis has occurred, the EPSs could be contaminated by proteins and pigments, but also other high-molecular-weight polysaccharides such as storage carbohydrates (starch or glycogen). These classical methods will then fail in separating the two polysaccharides. Even if the extraction method is conducted carefully, these contaminations are frequently encountered, as discussed by [191], and described for *Thalassiosira pseudonana*, *Phaeodactylum tricornutum*, *Cylindrotheca fusiformis*, *Craspedostauros australis*, *Pinnularia viridis* [201], *Navicula phyllepta*, and *Nitzschia epithemioides* [100], but also *Navicula jeffreyi* [202], or *Cylindrotheca closterium* [100,203]. As most of the EPSs from microalgae and cyanobacteria are anionic polymers, due to the presence of uronic acids and/or sulfate groups, the method developed by [143] for the extraction of the cell-bound polysaccharide from *Porphyridium* can be efficiently used. Briefly, after the hot water treatment, the polysaccharide is selectively precipitated using the cationic compound cetyl pyridinium chloride, converted to its calcium salt, and reprecipitated with ethanol. Some variants of this protocol have been implemented since. The authors of [40] have proposed a method to extract BPSs from *Arthrospira platensis*. First, during the hot treatment, the biomass is suspended in a TAPS buffer (0.05 M), and supplemented with EDTA (0.025 M) and NaCl (0.025 M) to limit the cellular lysis. After centrifugation, the supernatant containing the polymer is precipitated by the addition of 10 volumes of 3% cetyl trimethyl ammonium bromide (CTAB), which is assumed to allow the specific precipitation of acidic polysaccharides. After a new precipitation step, the pellet is dissolved in 1 M KCl before precipitation with ethanol. These latter solubilization/precipitation steps were repeated two times with decreasing KCl concentration solutions (0.75 and 0.3 M) in order to destabilize the complex between the CTAB and polysaccharides. The same protocol was successfully applied to *Porphyridium* by [130], as well as for *Chroodactylum ornatum*, *Chroothece richteriana*, *Bangiopsis subsimplex*, *Rhodaphanes brevistipitata*, and *Rhodospora sordida* [32]. In addition, an ultrafiltration step can be added at the end of the protocol to remove remaining salts, allowing the purity of samples to increase [32]. However, even if this method allows the efficient extraction and selective purification of the anionic cell-bound PSs, the high number of successive steps makes the method hardly scalable.

Some authors have also described the use of fixative agents such as formaldehyde or glutaraldehyde that can chemically react with hydroxyl, sulfydryl, carbonyl, or amino groups of the outer membrane, to prevent cell lysis during extraction [204,205]. An advantage is then that the PSs could be further recovered by classical ethanolic precipitation. However, some chemical modifications of the EPSs were noticed [197,202,206,207].

A few studies have also dealt with treatments using cationic resins. These resins have been shown to disturb the interaction of BPSs with the cellular membrane without cellular lysis, allowing their recovery [202,207]. Although this method seems promising, regarding the purity and the extraction yields that can be reached, its use is actually not possible at an industrial level due to the high cost of resins and the low amount of the sample that can be treated, limiting its application to a structural analysis purpose.

Finally, in a biorefinery context aiming for the co-valorization of several compounds, the study of [189] allowed the sequential purification of BPSs and pigments (B-phycoerythrin, B-PE) from *Porphyridium cruentum*. In this work, the authors used a high-pressure treatment to achieve cellular lysis and the release of B-PE, with, as a consequence of the mechanical constraints, the simultaneous release of BPSs. They then developed a two-step ultrafiltration process, using successively 300 kDa and 10 kDa MWCO polyethersulfone membranes, to further purify both fractions.

## 5. Applications

### 5.1. Biological Activities

The EPSs produced by microalgae and cyanobacteria have been the subject of numerous publications regarding their biological activities. This includes, among others, antioxidant, antiviral, antifungal, antibacterial, anti-ageing, anticancer, and immunomodulatory agent. This part has, therefore, no claim to be exhaustive regarding the huge number of available studies.

Many EPSs from microalgae and cyanobacteria have been shown to exhibit antioxidant activities (scavenging abilities on superoxide radicals, hydroxyl radicals, and hydroxyl peroxide) including *Spirulina platensis* [208,209], *Porphyridium* sp., *P. cruentum*, *Rhodella reticulata* [210,211,212,213], *Schizochytrium* sp. [214], *Isochrysis galbana* [215], *Nostoc carneum* [216], as well as *Scenedesmus quadricauda*, and *Chlorella vulgaris* [88]. In all cases, the activities were shown to be dose-dependent.

Antibacterial and antifungal properties have also been described, with strong variations in the minimum inhibitory concentrations (MIC) from a polymer to another, and from a bacterial strain to another. EPSs from *Gloeocapsa* sp., *Rhodella reticulata*, and *Synechocystis* sp. inhibited the proliferation of *Staphylococcus aureus*, with MICs equal to 0.125, 0.25, and 1.00 mg mL^−1^, respectively [118]. For the same bacterial strain, the EPS from *Porphyridium marinum* was found at least as efficiently (MIC of 0.125 g L^−1^) and has also shown an antibacterial effect on *E. coli*, *Salmonella enteritidis*, and, to a lesser extent, on *S. aureus* Methicillin Resistant (1 g L^−1^) [217]. In addition, the EPS from *Gloeocapsa* sp. was the only one inhibiting the growth of the fungus *Candida albicans*, while the one from *Synechocystis* sp. inhibited the growth of *Pseudomonas aeruginosa* [118]. If the EPS from *P. marinum* was found to be unable to affect *Candida albicans* growth, in contrast, it can prevent biofilm formation, with an efficiency similar to farnesol, which was used as a control (90% inhibition at 31.3 µg L^−1^) [217].

Antiviral activities are probably the most studied biological activities for EPSs from microalgae and cyanobacteria, even if the mechanism is still not well understood. This is the case of the EPS isolated from *Cochlodinium polykrikoides* which has antiviral properties in vitro against the Respiratory Viruses (RSV-A and RSV-B) and influenza virus types A and B [89]. In addition, the EPS inhibits the replication of the HIV-1 virus at a concentration IC50 equal to 1.7 μg mL^−1^ [89]. The sulfated EPS from *Porphyridium* sp. also has an antiviral effect against the herpes virus (HSV-1 and HSV-2), with an optimal concentration of about 100 μg mL^−1^ [218,219], whereas the sulfated polysaccharide p-KG03 isolated from *Gyrodinium impudicum* has a strong activity against the encephalomyocarditis virus (EMCV) [30]. Finally, the EPS from *Arthrospira platensis* has shown strong inhibiting activity against human cytomegalovirus, herpes simplex virus type 1 and 6 (HSV-1, HSV-6), and human immunodeficiency virus type 1 (HIV-1), while only weak or no inhibition was noted for the Epstein–Barr virus and influenza A virus [220,221,222].

Some antitumor activities have also been highlighted. In most cases, the activity has been related to the immunomodulatory effect of polymers. Indeed, the EPS from *Porphyridium cruentum* improves the immune response by stimulating the proliferation of macrophages and lymphocytes. These activities have been found to be greater for small molecules (Mw = 6.55 × 10^4^ Da) in comparison to larger ones (Mw = 2.56 × 10^5^ Da) [223]. Indeed, in the study of [217], an antiproliferative effect of EPSs from *P. marinum* was noticed on mammary carcinoma cells, with an increasing efficiency with the decrease in molar mass. The homopolysaccharide from *G. impudicum* inhibited tumor cell growth both in vitro and in vivo [224]. In vivo studies were also conducted with EPSs from *P. cruentum*, administered to shrimps. A non-specific immune response was evaluated and increases in the total hematocyst value, phagocytic activity, and respiratory burst were noticed as a function of EPS concentration [225]. The EPS from *Tribonema* sp. was found to stimulate macrophage cells, upregulating interleukin 6 (IL-6) and 10 (IL-10), as well as the tumor necrosis factor (TNF). The anticancer activity on HepG2 cells was found to be dose-dependent, with an inhibition rate of 66.8% for 250 µg mL^−1^. Additionally, this anticancer activity seems to mainly induce cell apoptosis, rather than affecting the cell cycle and mitosis [104]. The increase in the level of pro-inflammatory cytokines TNF-α and IL-6 was also noticed for the EPS from *Nostoc* sp., jointly with a release of prostaglandins and nitric oxide, via the induction of COX-2 and iNOS expression, respectively, suggesting an effect on the early innate immune response [226]. However, the EPSs from microalgae can also be used as anti-inflammatory agents. This is the case of the polymer isolated from *Porphyridium* sp. which inhibits the migration of leukocytes to the site of inflammation [227].

Finally, some other biological activities have been demonstrated, such as the specific reduction of the frequency and intensity of the cough without avoiding the expectoration by the EPS of *Rhodella grisea* [228]. This interesting effect was also noticed for the EPS from *Nostoc* sp., orally administered to guinea pigs. At the dose of 75 mg kg^−1^, the bronchodilatator effect, monitored by the reduction of the cough effort and the airway reactivity, was found to be similar to codeine (antitussive drug, dose 10 mg kg^−1^) and salbutamol (antiasthmatic drug, 1 mM solution) [226]. Finally, anticoagulant activity was demonstrated for the EPS from *Arthrospira platensis* [53], while the EPSs from *Rhodella violacea*, *Rhodella maculata*, *Porphyridium marinum*, and *P. purpureum* have shown an in vitro activity towards *Encephalitozoon cuniculi* microsporidia and in vivo activity against *Nosema ceranae* [106]. This anti-parasitic activity can give rise to their use in the treatment of nosema, a disease contributing to honey bees’ mortality.

Despite the numerous studies demonstrating the biological activities of EPSs from microalgae and cyanobacteria, the mechanisms by which they act remain almost unclear. As these polymers are frequently sulfated, they may act as mimetics of glycoaminoglycans (GAG). These GAGs (heparin/heparan sulfate, chondroitin sulfate, dermatan sulfate, keratan sulfate, hyaluronan) constitute a family of polymers with a high sulfate content (except hyaluronan) and are major components of the extracellular matrix in animal tissues, where they are implied in many biological functions. Many authors have claimed that EPS activity is directly linked to the sulfate content of the GAGs, as they may interact with proteins, such as receptors or growth factors [229,230,231,232]. Thus, for the EPS from *Porphyridium*, [11] confirmed that there was a linear relationship between the sulfate level in the polysaccharide and its antiviral activity against the Herpes virus. For the specific case of antiviral activities, several mechanisms have been proposed, related to the replication of the viruses during the early stages [233,234], or inhibiting the virus penetration [220,221] by competing with the virus for the attachment sites [235,236,237,238]. However, the action mechanism could be different depending on the biological activity tested, and it remains very difficult to rely on a structure and a function. For instance, the level of sulfation appears to not always be a sufficient parameter to consider, as shown for antiparasitic activity [106]. These authors suggested a possible implication of the position of the sulfate groups, as several polysaccharides from algae were tested, and the more active one was not the more sulfated. While testing the antitumor activity of polysaccharides from the seaweed *Gayralia oxysperma*, the same conclusion was obtained [239]. More than the sulfate presence, it seems that the anionic behavior of the polysaccharide could be of importance in some cases, as some polymers exhibiting uronic acids in their structure (but no sulfate groups) were shown to be active, hyaluronic acid being one of the most extensively studied examples. Moreover, the implication of some specific monosaccharides in the EPS composition could also be discussed. As an example, and even if this assumption is still under discussion, some biological activities described for the fucans may be in relation to their high content in fucose. Indeed, polysaccharides from 8 green algae species, with similar sulfation levels, have been tested for their anticoagulant activities [240]. The arabinose-rich polysaccharides exhibited a potent thrombin inhibition effect (even stronger than that of heparin), followed by galactose-rich ones. All these findings suggest also that the operating parameters chosen to improve EPS production yields are of importance, depending on the targeted application, as the composition of the polymer could vary, as previously discussed. Finally, other parameters should have an implication on the biological activities (molecular mass and branching pattern of polysaccharides, type of activity tested, acting mechanism, and so on), explaining the difficulty to establish a clear scheme for the structure–function relationship.

### 5.2. Physico-Chemical Properties

PSs from bacteria or higher plants (including seaweeds) are commonly used as thickeners, stabilizers, and gelling agents in the agri-food, pharmaceutical, and cosmetics industries [241]. EPSs from microalgae and cyanobacteria could present interesting physico-chemical properties, making them promising for such applications. The main limiting factor for their exploitation is actually the production costs that are still not competitive with PSs from other sources.

Several studies have been conducted on the flow properties of EPSs, in solution, from microalgae and cyanobacteria [105,148], but unfortunately, these have been limited to a really low number of species. Concerning microalgae, all works were focused on EPSs from red microalgae such as *Rhodella* or *Porphyridium*. As an example, dilute solutions (0.25%, *m*/*v*) of EPSs produced by red microalgae presented viscosities between 23.5 and 27.5 cP for the polysaccharide of *Porphyridium* sp., and equal to 28.7 cP for the polymer of *Rhodella reticulata* [242]. Similar results were obtained with the EPSs from *Porphyridium cruentum*, for which the viscosity was found significantly higher when the EPS was extracted from cultures containing 21 mM of MgSO_4_ or MgCl_2_ [105]. Solutions of these microalgae EPSs all presented a non-newtonian behavior [210,243], with the viscosity decreasing with the increase of the shear rate, which is typical of a shear-thinning comportment [244]. This decrease in viscosity with the shear rate has been suggested as being linked to the dissociation of the strong hydrogen bonds existing between the chains of EPSs [245]. The partial depolymerization of EPSs from *Porphyridium*, has been shown to strongly affect viscosity, with, for the lower molecular weight samples, a comportment typical of Newtonian fluids [210]. This finding confirm that a high viscosity is correlated (at least partly) to the high molecular mass of EPSs. Recently, [22] studied the rheological properties of RPSs and BPSs (2% *w*/*w*) from *Porphyridium cruentum*. The RPSs possessed high intrinsic viscosities in comparison with those described for commercial thickeners, such as alginates, locust bean gums, and guar gums, but lower than those of xanthan gums [246,247,248]. A lower intrinsic viscosity was observed for BPSs, which is not surprising as the molecular weight of the polymer was found to be lower. Nevertheless, the value obtained was still in the range of some commercial thickening agents, confirming that both fractions could be promising for use in the hydrocolloid market. While studying physico-chemical comportments in oscillation mode, some differences were highlighted. For RPSs, a predominantly elastic behavior was observed, since values for the storage modulus (G′) exceeded those of the loss modulus (G″). However, no true gel was observed (ratio G″/G′ should have been in the order 10^−2^), but they clearly presented a weak gel behavior. A similar finding was made by Medina-Cabrera 2021 while comparing EPSs from *P. purpureum* and *P. sordidum*. In contrast, viscous behavior was observed for BPSs, with a constant loss modulus and G′ < G″ over the whole range of shear strains explored. This fraction can then be assimilated to a viscoelastic fluid [22]. Furthermore, the rheological properties of EPSs from *Porphyridium* were found stable in a wide range of pH values (2–9) and temperatures (30–60 °C) [249,250]. However, contrasting observations were reported by different authors when temperatures were further increased. Three gelation mechanisms are well documented for polysaccharides: (i) ionotropic gelation (typically cation-mediated gelation), (ii) cold-set gelation, and (iii) heat-set gelation. The authors of [244] observed a strong gelation at temperatures above 60 °C which was reversible upon cooling (thus belonging to the last category), whereas other authors reported the rheological properties to be independent of temperature [190,250]. Regarding the impact of temperature on the rheological properties of EPS from *Porphyridium*, attention also has to be paid to the drying process used as a post-production treatment, as [251] have shown that a temperature > 90 °C may cause significant alterations to the polymer, and thus affect the intrinsic viscosity and elasticity properties. As these polymers are anionic, due to their content in uronic acids and sulfate groups, it has been suggested that the high viscosity values and the weak gel behavior observed can be linked to an interaction with cations, leading to a partial cation-mediated gelation [244,252]. However, no increase in viscosity was observed by [22] in the presence of different cations, and this behavior has been recently confirmed by [249] for EPSs from *P. purpureum* and *P. sordidum* while adding NaCl. By using a divalent cation (CaCl_2_), these authors even noticed a decrease in viscosity for both polymers.

EPSs from cyanobacteria also possess interesting rheological properties. For example, the production of EPSs by *Cyanospira capsulata* induced a viscosity increase of the culture medium. After 31 days of batch culture, the viscosity has been measured around 450–500 cP [253]. The viscometric analysis of supernatants collected at different times of culture has revealed a typical pseudoplastic behavior [253]. An increase in the culture medium viscosity was also observed for *Synechococcus* sp. The viscosity was mainly due to the EPSs, as viscosity measurement results were similar for solutions with or without cells [128]. The rheological study has shown that the viscosity decreased when the shear rate increased, reflecting once more a shear-thinning behavior. *Anabaena* sp. EPSs also presented this typical property in diluted solutions (0.6% *w*/*w*) [147]. Similar results were obtained on several EPSs isolated from *Cyanothece*, even at a lower concentration (0.1% (*w*/*w*) [148]. For 1% (*w*/*v*) EPS solutions from *Nostoc flagelliforme*, the apparent viscosity was found to be intermediate, between 0.5% and 1% (*w*/*v*) xanthan gum solutions, but the decrease observed with a shear rate was faster, leading quickly to low viscosity solutions (two times less than the viscosity of the 0.5% xanthan gum solution). The difference in viscosity with xanthan gum was attributed by authors to a lower molecular mass of EPS, which was about 2 × 10^5^ Da for EPSs and 2 × 10^6^ Da for xanthan [254]. For EPSs from *Nostoc carneum*, shear-thinning behavior was also observed, but the viscosities reported were found to be lower, and requested a 15 mg mL^−1^ solution to obtain a viscosity of about 140 cP [216]. The same kind of comportment was described for EPSs from *Arthrospira platensis*, requesting a high polymer concentration (5% *w*/*w*) to observe a significant viscosity [255]. At this concentration, solutions were found to present a linear viscoelastic range (G′ and G″ are constant and G′ > G″), indicating a viscoelastic gel-like behavior [255].

In addition to these rheological properties, the anionic nature of the EPSs confers some interesting affinity towards cations. Thus, some EPSs may present some gelation capacity similar to alginates or pectins PSs. The low number of EPSs from microalgae and cyanobacteria studied for their physico-chemical properties has not yet allowed the identification of this kind of gelation ability, but a lot of the original comportment remains to be discovered. The chelation ability of EPSs will not be developed here, but has already been described for several EPSs, mainly from cyanobacteria. Such a property may open the way to the industrial use of EPSs for biosorption and water treatment to remove heavy metals [256,257,258,259].

Aside from their hydrophylic nature, EPSs can also present significant hydrophobic behavior, in relation to ester-linked methyl or acetyl groups, together with peptidic moieties [78,260]. The presence of both hydrophilic and hydrophobic groups can suggest the potential utilization of the polymers as bioflocculants [254], biosurfactants, or bioemulsifiers [261]. This kind of study is rather scarce, but the use of EPSs from *Nostoc flagelliforme* for the stabilization of emulsions has been demonstrated, with, depending on the oils used, emulsification activities similar to xanthan gum [254].

### 5.3. Other Properties: Plant Stimulating Action

Apart from biological activities and rheological properties that may lead to applications in pharmaceutical and food markets, an increasing interest is on the use of microalgae polysaccharides for use as plant biostimulants. Plant biostimulants are substances whose function, when applied to plants, is to stimulate natural mechanisms to enhance plant growth, nutrient use efficiency, and tolerance to abiotic stressors. Seaweeds have been largely studied for the production of plant growth biostimulants [262,263,264], including focusing on polysaccharides, but studies on microalgae are much more recent, and often dealing with complex microalgae extracts instead of purified molecules. Indeed, only in the last decade have the effects of microalgae EPSs on the growth and development of several crops been studied. Microalgae polysaccharides can stimulate plant growth and nutrient uptake by modulating various physiological and biochemical processes. The authors of [265] showed that polysaccharides from *S. platensis* significantly promoted plant growth in *Capsicum annuum* (pepper) and *Solanum lycopersicum* (tomato), which was demonstrated in terms of plant weight, plant size, and size/number of leaves. The application of 1 mg mL^−1^ EPS solution from *A. platensis*, *D. salina*, and *Porphorydium* sp. on *Solanum lycopersicum* plants significantly improved the nodes number, shoot dry weight, and shoot length, without strong differences between the polymers [266]. Crude extracts rich in polysaccharides from *Scenedesmus* sp and *A. platensis* showed a good capacity for improving plant growth, development, and the leaf nutrient status of the Petunia x hybrida plant [267]. Another study showed that liquid extracts of *C. vulgaris* and *S. quadrica* induced the stimulation of the root development of *Beta vulgaris L.* plants [268], while [269] showed that exopolysaccharides extracted from *P. tricornutum* and *D. salina* stimulated the germination of pepper under salt stress conditions. The potential of *D. salina* EPSs to attenuate the effect of salt stress has also been evaluated on *Solanum lycopersicum*, showing that the application of the polymer can mitigate the decrease in length and dry weight of the plant shoot and root systems, but also activate/inhibit various metabolic pathways involved in the plant’s tolerance to stress, such as jasmonic acid-dependent pathways [270]. Additionally, this EPS can improve the seed germination and seedling growth of *Triticum aestivum L.* (wheat) under salt stress [265].

The direct relationship between the molecular structure of microalgae EPSs and their biostimulant activity is yet to be understood, but the greater knowledge about the effect of seaweed polysaccharides could probably contribute to the elucidation of the general mechanisms. During their entire life cycle, plants are exposed to several environmental stresses, which negatively affect their productivity and survival. One of the most common indications of stress is the production of a reactive oxygen species (ROS). Interestingly, it has been demonstrated that microalgae EPSs can mitigate ROS toxicity in plants by enhancing the production of ROS antioxidant enzyme activities. This mechanism can be explained by the fact that EPSs may interact with plasma membrane receptors as signal molecules, thereby leading to the activation of a series of biochemical reactions. In his study, [266] demonstrated that the application of EPSs from *D. salina* enhanced the accumulation of proline and the ROS antioxidant enzyme activities of Catalase (CAT), Peroxidase (POD), and Superoxide dismutase (SOD) in plants subjected to saline stress. The authors of [271] also reported that treatment with *C. vulgaris* polysaccharides stimulated the enzymatic activity of Ascorbate Peroxidase (APX) in tomato plants. In the same study, microalgae polysaccharides extracted from *C. reinhardtii* and *C. sorokiniana* significantly enhanced POD activity.

Apart from environmental stresses, plants could also be the target of pathogen attacks, and microalgae EPSs could be promising as elicitors to contribute to plant defense against those pathogens. The authors of [271] indicated that microalgae crude polysaccharide extracts can induce plant innate immunity, depending on the microalgae strain. In his study, plants treated with polysaccharides extracted from *C. vulgaris* and *C. sorokiniana* exhibited a significant increase in β-1,3-glucanase activity, which is one of the pathogenesis-related proteins, grouped in the PR-2 family that break down the cell wall components of pathogens. The induction of PR proteins is a consequence of the activation of plant defensive pathways, which limit the entry, or the further spread of the pathogen [272]. In the same study, *C. sorokiniana* crude polysaccharides had a significant stimulatory effect on Phenylalanine ammonia lyase (PAL) activity, indicating an upregulation of the phenylpropanoid pathway, leading to the production of toxic molecules against the pathogens [273].

### 5.4. Potential Commercial Markets

**Figure 3:**
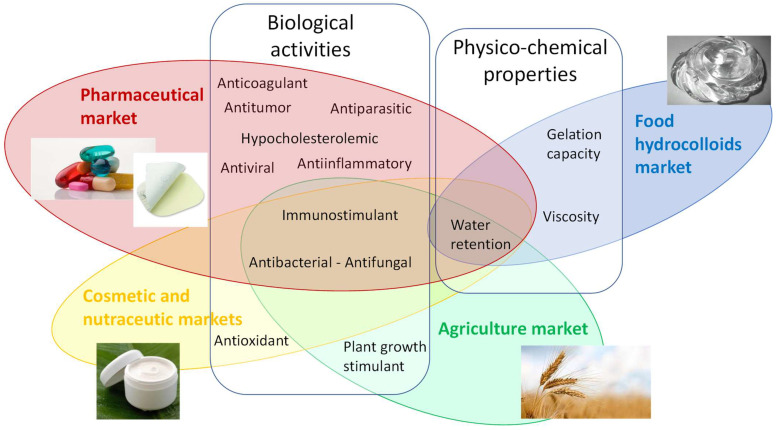
Interconnection of the EPS biological activities, physico-chemical properties, and the potential commercial markets.

Taking into account their properties, the valorization of EPSs from microalgae and cyanobacteria can be considered in the areas of food, cosmetics, pharmaceuticals, and agriculture. The same property can be of interest for several applications, depending on the targeted product, while others are more specific and related to only one potential market. This is illustrated on Figure 3, showing the interconnection of the EPS properties and the potential commercial markets. However, only a very few numbers of commercial EPSs can actually be found, and they are mainly in the cosmetics industry. Their valorization in the other areas (therapeutic, human food and animal feed, nutraceuticals) is very reduced, mainly because of the too-high production costs and the lack of information on their structure. The polysaccharide market has a constant growing rate, evaluated between 1–3% in 2011 [274]. Indeed, the market could assimilate some polysaccharides from microalgae with original biological or technofunctional properties. Even if some technological bottlenecks subsist, the microalgae technologies (production, harvesting, biorefinery) have been significantly improved in recent decades. The autotrophic production of microalgae could reach the breakeven point with high cell density cultivations and their treatments by specific downstream processes, opening them up to low value markets, such as the food industry. If, despite the efforts and technical improvements, the production cost of EPSs remains high, their valorization will be limited to the high-value molecules markets, such as those of the cosmetic or therapy markets.

The next section deals with a few examples of the use of EPSs in various industrial areas, mainly for EPSs from red microalgae or from *Arthrospira platensis*.

#### 5.4.1. Cosmetic and Nutraceutic Markets

As previously said, this market is the most developed for the use of EPSs from microalgae and cyanobacteria, mainly for their biological activities, and especially their anti-inflammatory, antimicrobial, and antioxidant activities (Figure 3). They are also described as moisturizing agents, as they are anti-ageing and strengthen the barrier function of the skin [211,275]. This moisturizing effect is linked to the strong water retention capacity of EPSs, related to the fact that EPSs are supposed to be produced to support desiccation [8,276]. As an example, the moisture retention capacity of EPSs extracted from the cyanobacterium *Aphanothece sacrum*, is 10-fold higher than hyaluronic acid [277,278]. However, the market is surprisingly mainly occupied with *Porphyridium* EPSs, despite the many other EPSs that can have interesting applications in this field. An exception is a mixture of polysaccharides extracted from a *Chlorella* strain grown heterotrophically [279], patented by the Solazyme company and commercialized with the registered trade name ‘Alguronic Acid’. Ref. [280] has developed a cosmetic formulation containing EPSs from *Porphyridium*, allowing them to repair and strengthen the skin barrier. Previously, the L’Oréal company had patented a mixture of EPSs from *Porphyridium* sp., of a marine bacterium and ulvan (a polysaccharide from the seaweed *Ulva lactuca*), associated with a C-glycoside [281]. They claimed that this active ingredient can improve the barrier function of the skin, as well as its hydration. This same company has developed another patent based on the use of the EPSs to eliminate and/or reduce the dandruff of the scalp, as an alternative to the zinc pyrithione frequently incorporated into shampoo [275]. The same EPSs from *Porphyridium cruentum* have been tested on three enzymes (hyaluronidase, elastase, and collagenase) involved in the degradation of the three respective substrates, hyaluronan, elastin, and collagen, which are key compounds for the appearance of the skin [282]. Even if no activity against collagenase was noticed, the EPSs strongly reduced the activity of the hyaluronidase at concentrations between 0.25 and 2.5 mg mL^−1^ (96.6 ± 0.3%), as well as the activity of elastase at a concentration of 5 mg mL^−1^ (46.0 ± 7.1%). These results have been patented by the company Solazyme [283] for use in cosmetic formulations. Recently, EPSs from several microalgae (*Phaeodactylum ornatum*, *Synechococcus*, *P. cruentum*, *Glossomastix* sp., *Chrysotila dentata*, *Pavlova* sp., *Diacronema* sp.) have been patented as anti-ageing agents. Indeed, these EPSs were shown to stimulate collagen and hyaluronic acid production, with a greater activity while decreasing molecular mass [284]. Nowadays, several companies propose these cosmetic formulations or active ingredients, such as Frutarom, Asta technologies, and Yemoja, all three located in Israel, Algosource technologies (France), Micoperi Blue Growth (Italy), and Solazyme (USA).

Concerning the food and nutraceutic markets, the *Porphyridium* EPS is once more the most represented, even if some *Arthrospira* (spirulina) extracts can be found. However, in this latter case, as *Arthrospira platensis* is allowed for food consumption in its whole biomass form, the EPS is generally not extracted and purified. Nutraceuticals correspond to biomolecules having a positive effect on health that can be incorporated into food. This market has been in constant growth for twenty years, as shown by the exponential increase in the number of publications on the subject [285].

Ref. [286] has shown that EPSs from *Porphyridium cruentum* are not hydrolyzed in the gastrointestinal tract. Therefore, their bioavailability is null, and, associated to the high viscosity of EPS solutions, their use as dietary fibers could be of interest. Moreover, [287] have demonstrated a hypocholesterolemic effect (on a rat model) of EPSs from *Porphyridium* sp. Feeding rats with this EPS were found to significantly improve levels of total serum cholesterol, serum triglycerides, hepatic cholesterol, HDL/LDL ratios, and increased fecal excretion of neutral sterols and bile acids. The nutraceutical use related to these biological activities was patented by the company Solazyme in 2007 [288]. The same year, the Solazyme company also developed a nutraceutical composition with an antioxidant activity from the same EPS [289]. This formulation allows the reduction of inflammation and prevents oxidative damage in the tissues of mammals. Finally, [290] have shown that the EPSs from *Porphyridium* could be used to reduce the blood glucose level in mice.

#### 5.4.2. Pharmaceutical and Medical Markets

As previously described, EPSs from microalgae and cyanobacteria present numerous biological activities that may allow their use as pharmaceutical drugs. However, to our knowledge, such compounds are still not on the market, despite the fact that some patents can be found. EPSs from *Porphyridium* sp. have been patented as antiviral agents [291] or for the treatment of arthritis [292], whereas EPSs from red microalgae can be used for veterinary purposes, such as an antiparasitic agent to prevent the infection of honey bees by microsporidia [293]. The reason why no EPSs are on the market at this time is not only linked to economic reasons, but rather to the drastic legislation of therapeutic compounds. Before the exploitation of these EPSs, biological activities at a lab scale should first be confirmed by clinical trials that remain to be conducted. A strict control of the production systems, purity, stability, and safety are required, implying that some processes still require development. However, the main scientific lock to their exploitation is certainly the lack of detailed structured information, together with difficulties of identifying the mechanism of action. The road to the commercialization of new efficient drugs from microalgae will still be long and challenging.

The pharmaceutical application, that appears to be faster in its development, can use EPSs in wound healing bandages. Such hydrocolloid dressings can absorb and retain liquid exuding from the wound, prevent bacterial infection, and help healing. They have been developed and are commercialized with polysaccharides such as hyaluronan, gelatin, or alginates [294,295]. The high water retention capacity of EPSs from microalgae and cyanobacteria, associated to their antibacterial activity and anti-inflammatory effect, is then promising for such a market. Recently, such wound dressings including EPSs from *Nostoc* sp. have been successfully tested in vitro, showing their ability to promote fibroblast migration and proliferation, which are key parameters in the regeneration process of the skin [296]. Moreover, in the study of [297], four EPSs from cyanobacteria (*Tolypothrix tenuis* and 3 *Anabaena* species) were tested for their hemostatic activity, the one from *Anabaena* sp. showing a significant reduction of clotting time. Such wound healing hydrogels could, thus, not be limited to the protection and reparation of skin injuries, but also to hemostatic dressings.

#### 5.4.3. Hydrocolloids

Actually, there is neither EPSs from microalgae nor cyanobacteria on the hydrocolloids market, despite their original physico-chemical properties. This time the reason is probably mostly of an economic nature. The hydrocolloid market is mainly driven by seaweed polysaccharides. The annual market for seaweed polysaccharides is around 9600 tons for agars, 26,500 tons for alginate, and 50,000 tons for carrageenans for a market value of around USD 1 billion [298]. However, the market demand is still in its expansion. Seaweeds are mostly harvested in the natural environment or cultivated in open ponds or in the sea. However, the development cycle of seaweeds takes one year, and harvesting is not possible during the whole year, even with sourcing all over the world. Moreover, the extraction yields and the composition (and then properties) of these seaweed PSs vary with the seasons and harvesting location. Probably to cope with the market request, overcome variability, and stabilize sourcing, the global demand on EPSs from bacteria (such as xanthan, gellan, or curdlan) have increased in recent years. In this context, EPSs from microalgae and cyanobacteria might be able to find their place in the future, on the condition of further reducing the costs of the production and downstream processes, making them competitive. A first patent has been recently published, proposing the use of EPSs from several microalgae species as “anti-settling” agents for food beverages, due to their weak gel behavior [299]. Nevertheless, many EPSs from microalgae have still not been characterized for their physico-chemical properties, and regarding the diversity of composition and structures, many different behaviors could be expected, with this originality opening the way to specific market demand.

#### 5.4.4. Agriculture Market

Due to the specific plant-stimulating action described previously, EPSs from microalgae can find a place in the agriculture market as biostimulants or elicitors of plant defense. In recent decades, there has been an increased demand for sustainably produced food, with less of a concentration on synthetic chemicals and a higher concentration on biologicals. Hence, biostimulants are helping to address this issue in a sustainable manner, by providing protection against stress and, thereby, stimulating the growth of the plant. However, until recently, the regulation in Europe was not really clear about these products, being sometimes classified among fertilizers, and other times being in the phytosanitary category, inducing delayed market launches. As a result, a large number of products were still not available commercially. The European regulation EU 2019/1009 has clarified this classification, and now biostimulant refers to products (microorganisms or substances) linked to biotic or abiotic stresses, while products improving the resistance against pathogens will be found in the biocontrol category. This clarification will, therefore, induce an increase of the market value, which was already about USD 3.2 billion in 2021. Even if there is actually no microalgae EPSs in the biostimulant and/or biocontrol markets, other poly- or oligosaccharides such as chitosan derivatives have already entered it, and those products could then find a place in the future.

## 6. Conclusions

Microalgae and cyanobacteria represent a huge taxonomic diversity as several hundred thousand species may exist. Only a limited number have been identified as EPS producers, and even less have been subject to studies for their characterization. Most of these EPSs have only been described for their monosaccharides composition, and the complexity of structural studies has, until now, limited their elucidation. At the moment, we are still at the beginning of the comprehension of factors affecting EPS production, and the diversity of comportment between strains, associated with the still expensive production/extraction processes, limit the cost-effective mass production. Furthermore, metabolic pathways involved in the production are still unresolved, and a better comprehension could open the way to greater production yields. Additionally, many biological activities and original physico-chemical properties have been described, allowing the consideration of these molecules in several industrial areas. Although some of these EPSs are already available in the cosmetics market, their entrance into the pharmaceutical market will still require long and hard work to secure production, purity, safety, and elucidate action mechanisms. Finally, a promising valorization area could be the hydrocolloids sector, which is in constant expansion and looking for atypical properties in answer to more and more complex food processes and elaborated products.

## Figures and Tables

**Table 8:** Partial structures of EPSs from microalgae or cyanobacteria.

Organisms	Structures	References
*C. augustae*	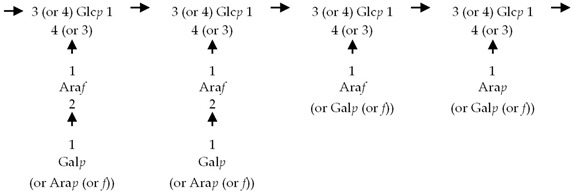	[77]
*C. corrosa*	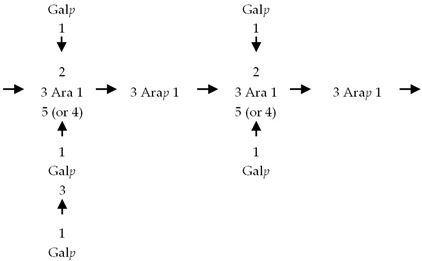	[77]
*A. densus*	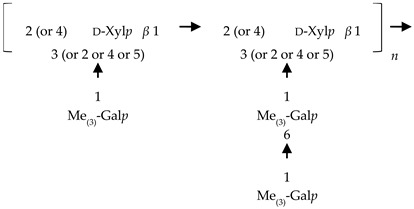	[73]
*P. cruentum*, *P. aerugineum*	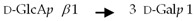	[58]
*Porphyridium* sp., *P. cruentum*, *P. aerugineum*, *R. reticulata*	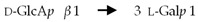	[129]
*Porphyridium* sp.	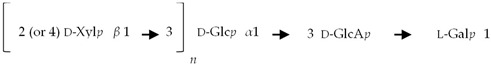	[108]
*Porphyridium* sp.	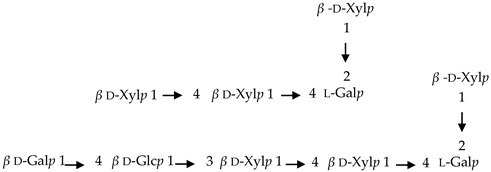	[130]
*Cyanothece* sp.	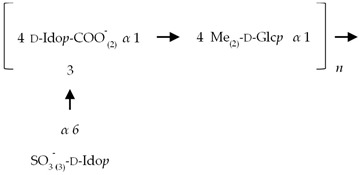	[131]
*C. capsulata*	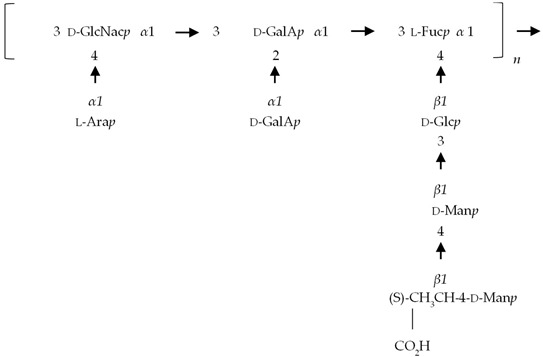	[132]
*N. commune*	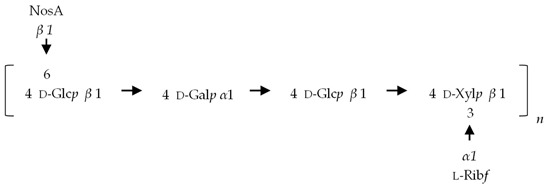	[124]
*N. insulare*	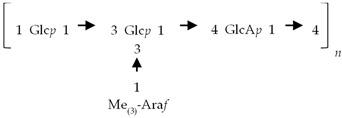	[60]
*O. planktothrix FP1*	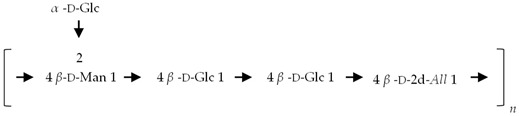	[127]
*M. laminosus*	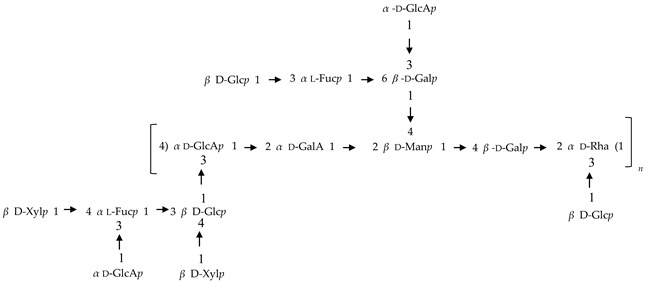	[21]
